# Comparing gene expression profiles of adults with isolated spinal tuberculosis to disseminated spinal tuberculosis identified by ^18^FDG-PET/CT at time of diagnosis, 6- and 12-months follow-up: classifying clinical stages of spinal tuberculosis and monitoring treatment response (Spinal TB X cohort study)

**DOI:** 10.1186/s13018-024-04840-7

**Published:** 2024-06-25

**Authors:** Julian Scherer, Sandra L. Mukasa, Karen Wolmarans, Reto Guler, Tessa Kotze, Taeksun Song, Robert Dunn, Maritz Laubscher, Hans-Christoph Pape, Michael Held, Friedrich Thienemann

**Affiliations:** 1 General Medicine & Global Health (GMGH), Department of Medicine and Orthopaedic Research Unit (ORU), Division of Orthopaedic Surgery, Faculty of Health Sciences, University of Cape Town, Cape Town, South Africa; 2 Department of Traumatology, University Hospital Zurich, University of Zurich, Zurich, Switzerland; 3 Department of Medicine, Faculty of Health Science, General Medicine and Global Health (GMGH), University of Cape Town, 4Th Floor, Chris Barnard Building, Anzio Road, Observatory, Cape Town, 7925 South Africa; 4 Institute of Infectious Diseases and Molecular Medicine (IDM), Department of Pathology, Division of Immunology, Faculty of Health Sciences, University of Cape Town, Cape Town, South Africa; 5 International Centre for Genetic Engineering and Biotechnology (ICGEB), Cape Town Component, Cape Town, South Africa; 6 Department of Medicine, CUBIC, PETCT, University of Cape Town, Cape Town, South Africa; 7 Division of Medical Microbiology, Department of Pathology, Faculty of Health Sciences, Institute of Infectious Diseases and Molecular Medicine (IDM), University of Cape Town, Cape Town, South Africa; 8 Orthopaedic Research Unit (ORU), Division of Orthopaedic Surgery, Faculty of Health Science, University of Cape Town, Cape Town, South Africa; 9 Department of Internal Medicine, University Hospital Zurich, University of Zurich, Zurich, Switzerland

**Keywords:** Tuberculosis, Spondylodiscitis, Spinal, Extrapulmonary, Musculoskeletal, Pott`s disease

## Abstract

**Background:**

Tuberculosis (TB) is one of the top ten causes of death worldwide, with approximately 10 million cases annually. Focus has been on pulmonary TB, while extrapulmonary TB (EPTB) has received little attention. Diagnosis of EPTB remains challenging due to the invasive procedures required for sample collection. Spinal TB (STB) accounts for 10% of EPTB and often leads to lifelong debilitating disease due to devastating spinal deformation and compression of neural structures. Little is known about the extent of disease, although both isolated STB and a disseminated form of STB have been described. In our Spinal TB X cohort study, we aim to describe the clinical phenotype of STB using whole-body ^18^FDG-PET/CT, identify a specific gene expression profile for different stages of dissemination and compare findings to previously described gene expression signatures for latent and active pulmonary TB.

**Methods:**

A single-centre, prospective cohort study will be established to describe the distributional pattern of STB detected by whole-body ^18^FDG-PET/CT and gene expression profile of patients with suspected STB on magnetic resonance imaging (MRI) at point of diagnosis, six months, and 12 months. Blood biobanking will be performed at these time points. Specimens for microbiology will be obtained from sputum/urine, from easily accessible sites of disease (e.g., lymph nodes, abscess) identified in the first ^18^FDG-PET/CT, from CT-guided biopsy and/or surgery. Clinical parameters and functional scores will be collected at every physical visit. Data will be entered into RedCap® database; data cleaning, validation and analysis will be performed by the study team. The University of Cape Town Ethics Committee approved the protocol (243/2022).

**Discussion:**

The Spinal TB X cohort study is the first prospective cohort study using whole-body 18FDG-PET/CT scans in patients with microbiologically confirmed spinal tuberculosis. Dual imaging techniques of the spine using ^18^FDG-PET/CT and magnetic resonance imaging as well as tissue diagnosis (microbiology and histopathology) will allow us to develop a virtual biopsy model. If successful, a distinct gene-expression profile will aid in blood-based diagnosis (point of care testing) as well as treatment monitoring and would lead to earlier diagnosis of this devastating disease.

*Trial registration*: The study has been registered on ClinicalTrials.gov (NCT05610098).

**Supplementary Information:**

The online version contains supplementary material available at 10.1186/s13018-024-04840-7.

## Background

### Epidemiology

**Fig. 1:**
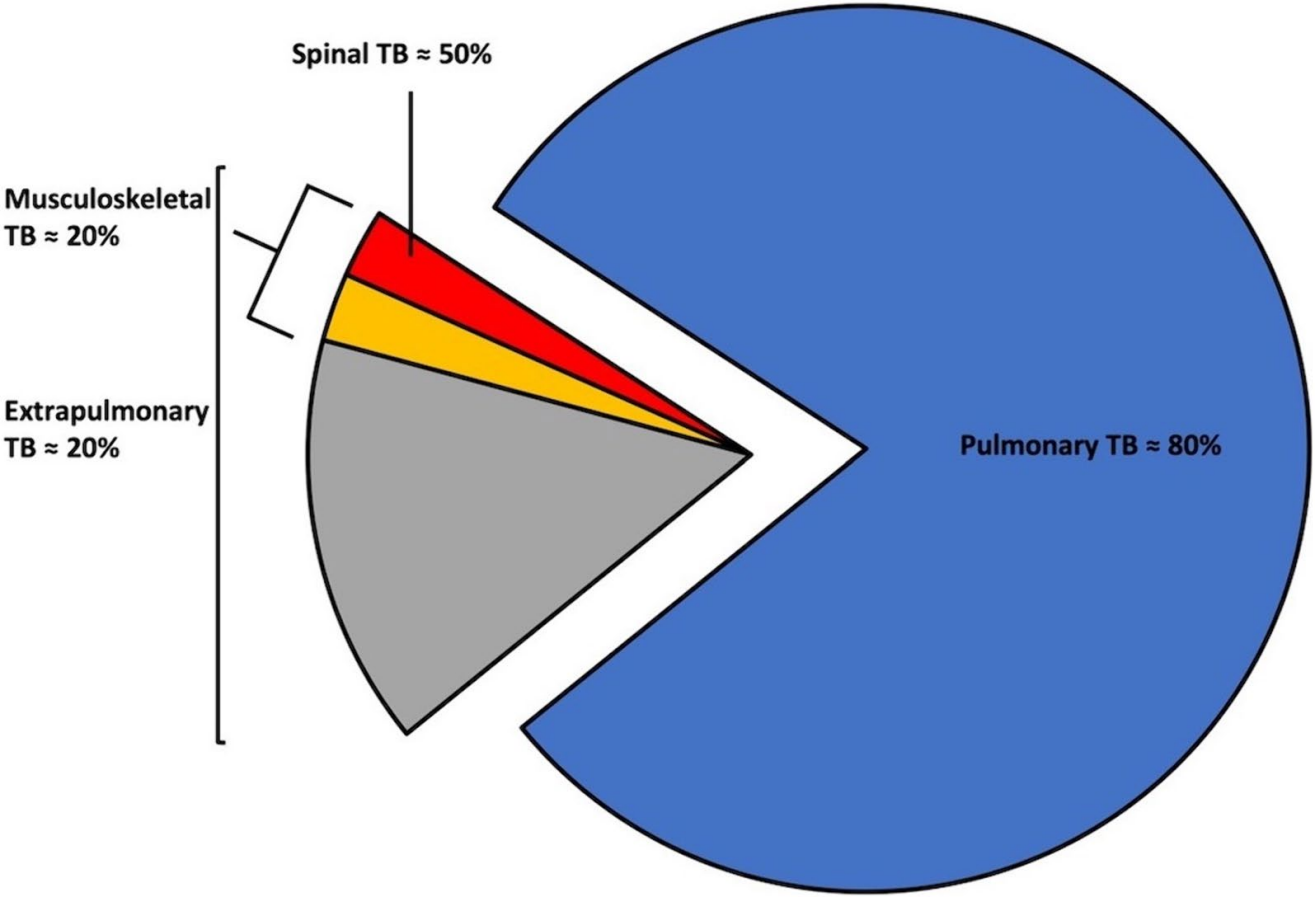
Pie chart depicting the proportions of different manifestations of tuberculosis (TB)

In 2022, an estimated 10.6 million people fell ill with tuberculosis (TB) and 6.4 million patients received TB treatment [1]. TB and HIV are the two leading infectious diseases causes of death worldwide, with TB ranking number one cause of death in people living with HIV (PLWH) and responsible for the burden of multimorbidity in low-and-middle-income countries [2–4]. South Africa has the most devastating TB/HIV epidemic with an HIV prevalence of 19% in adult TB cases and a TB incidence of 615/100,000 in some regions [5–8]. In 2019, 7.1% of the previously treated TB cases and 3.4% of the new TB cases in South Africa were resistant to rifampicin and/or isoniazid [9]. TB vaccine development has recently been successful, but not in PLWH who are more likely to develop extrapulmonary TB (EPTB) [10–12]. Musculoskeletal TB accounts for approximately 20% of EPTB cases, with spinal involvement in up to 50 percent of these cases (Fig. 1) [13, 14]. It is believed that spinal tuberculosis (STB) arises from the hematogenous spread of *Mycobacterium tuberculosis (Mtb)* from a primary pulmonary infection into the well perfused cancellous bone of the vertebral bodies. [15] STB is a spondylitis or spondylodiscitis caused by *Mtb*, historically called Pott's disease or Pott`s spine. It commonly manifests as localised backpain, pain, spinal deformity with/without instability, neurological deficit, and constitutional symptoms. The time from initial symptoms to diagnosis can take several years [16–18]. Diagnosis relies on clinical presentation, spinal imaging, and microbiological testing from lesion biopsy [19].Therapy includes TB treatment, immobilization, and spinal surgery in selected patients [20].

### Clinical and radiological phenotypes of spinal TB

**Fig. 2:**
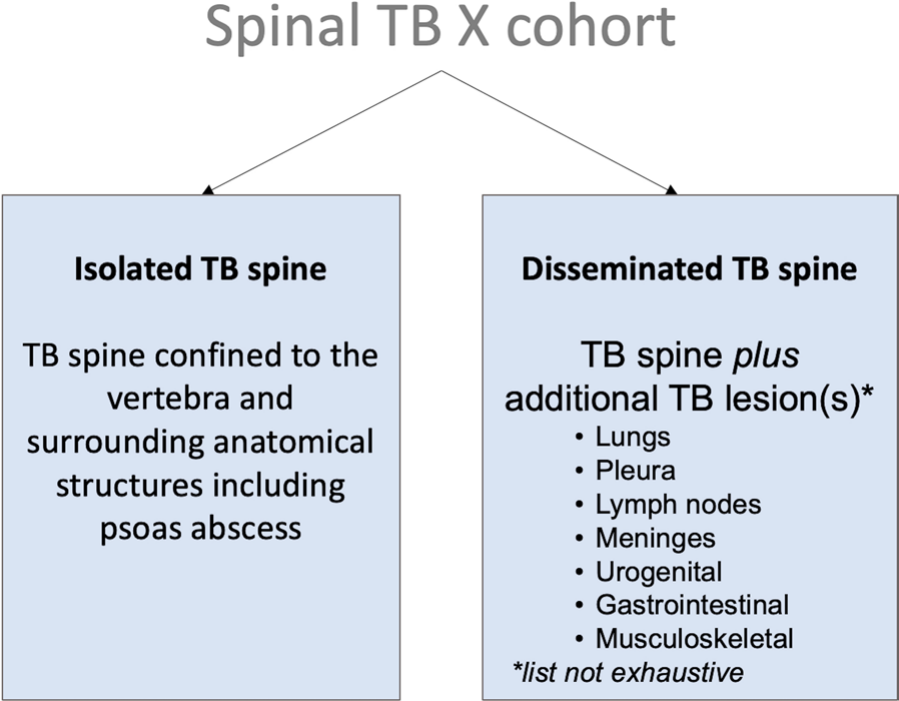
Definition of isolated spinal tuberculosis (TB) and disseminated spinal TB in the Spinal TB X cohort

**Fig. 3:**
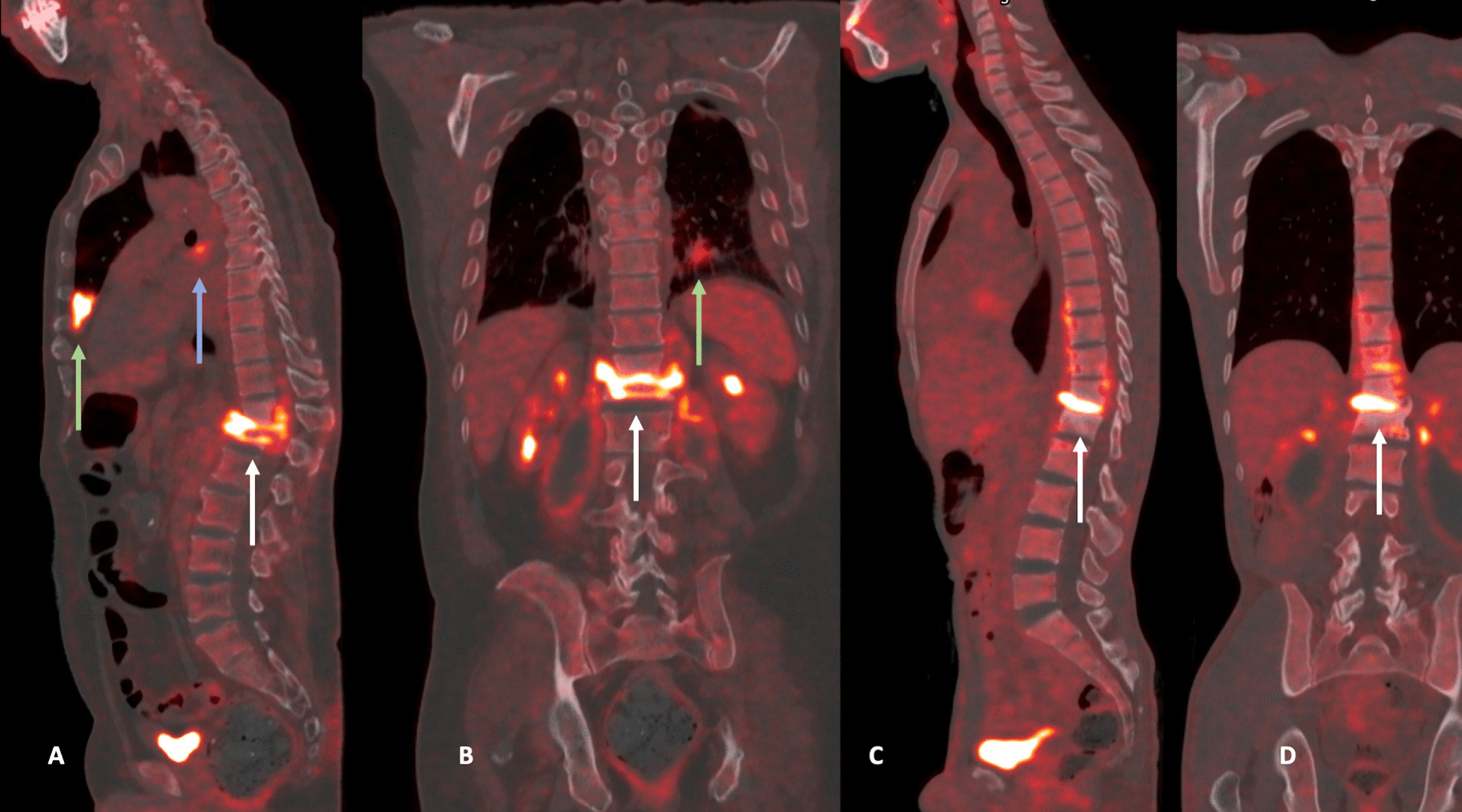
Example ^18^FDG-PET/CTs of two patients with spinal tuberculosis (TB). Patient 1 with sagittal (**A**) and coronal plane (**B**) with disseminated spinal tuberculosis. Spinal TB [white arrow] with a pulmonary TB lesion [green arrow] and mediastinal lymph node with FDG uptake [blue arrow]. Patient 2 with sagittal (**C**) and coronal plane (**D**) with isolated spinal tuberculosis. Spinal TB [white arrow] with no other lesion in the body in ^18^FDG-PET/CT

Little is known about the extent of disease in STB, and isolated STB, which is confined to the vertebra and surrounding anatomical structures, as well as disseminated forms of STB have been described (Fig. 2, 3) [21, 22]. The latter presents with spinal lesion(s) plus additional TB lesion(s) in other parts of the body such as the lungs, pleura, lymph nodes, meninges, urogenital, gastrointestinal tract, and other musculoskeletal involvement such as peripheral joints [23]. The diagnosis of disseminated STB requires whole body imaging and/or microbiological evidence of additional sites of infection, e.g., a positive GeneXpert from a lymph node aspirate or a positive urine culture for *Mtb* [24]*.* With this, the question arises, whether isolated spinal TB without any other active focus can be a stand-alone entity and whether the disseminated form of STB is a dual *Mtb* infection with potentially different strain types at the different sites of disease [25].

### Imaging in tuberculosis

Magnetic resonance imaging (MRI) is the imaging gold standard for STB and has a reported sensitivity of 94% and specificity of 93% in detecting spondylodiscitis [26]. MRI can accurately detect spinal cord compression, intrinsic changes of the cord, bony changes of the vertebral body as well as the extent of disc destruction, and psoas abscess formations [27]. If possible, MRI of the whole spine should be performed to detect non-continuous lesions, occurring in up to 20% of patients [28]. 2-Deoxy-2-[18F]fluoro-d-glucose positron emission tomography/computed tomography (PET/CT) has been shown to be able to detect sites of infection and monitor treatment response in tuberculosis [29]. ^18^FDG accumulates in tissue with increased glucose metabolism (e.g., infections, inflammatory conditions, malignant conditions, ischemia). It has been found that ^18^FDG uptake values are higher in patients with STB compared to patients with pyogenic spondylitis [30]. A small study has shown that ^18^FDG-PET/CT is potentially superior to MRI in accuracy to diagnosing STB [31]. Semi-automatic technique to quantify complex tuberculous lesions on ^18^FDG-PET/CT and allows for standardised assessment [32].

### Transcriptomic profiling in tuberculosis

Host transcriptomics have identified RNA signatures for tuberculosis diagnosis and monitoring treatment response as well as understanding the immunological mechanisms of pulmonary TB [33, 34]. Several RNA signatures have been identified for risk of disease stratification, screening for TB, tracking treatment response and prediction of treatment failure [35–38]. Lately, an RNA signature which predicts recent exposure to *Mtb* in humans has been identified [39]. The description of these RNA signatures is opening the possibility for developing large-scale point of care screening tests as well as targeted (personalized) intervention to prevent the disease and, in patients with active tuberculosis, create customized treatment plans [36]. However, the diagnosis of EPTB and particularly STB remains challenging simply because sample collection requires invasive procedures in the absence of a blood-based diagnostic test. Host transcriptomics may be the key for a blood-based test for STB.

### Treatment of spinal TB

Treatment combination and duration of treatment of STB has not been systemically studied in randomised controlled trials for various reasons. According to WHO, adults with extrapulmonary TB can safely be treated with a 6-month regimen except for patients with TB of the central nervous system and musculoskeletal TB, including patients with STB. These subgroups require 9–12 months of treatment duration.

Treatment for STB consists primarily of chemotherapy (antituberculosis treatment; ATT) and in case of a “spine at risk”, surgery is required additionally [40, 41]. Drug-sensitive TB can be treated using the standard combined 4-drug regimen medication according to body weight. The WHO suggests an intensive phase using with rifampicin, isoniazid, pyrazinamide, and ethambutol for two months, followed by continuation phase using rifampicin and isoniazid for seven to ten months [42] Guidelines for the treatment of multi-drug resistant (MDR) STB are lacking. According to WHO, patients with severe forms of extrapulmonary MDR TB including TB meningitis, brain abscesses, osteomyelitis and arthritis (which includes STB) should not be treated with the new 9-month all-oral regimen for MDR-TB and longer regimens apply for patients with severe forms of extrapulmonary diseases such as STB [43]. However, treatment durations of STB as well as dosing of TB drugs differ significantly between different countries and an infectious diseases expert with experience in extrapulmonary TB disease should always be consulted [44].

## Methods and design

### Aims and objectives

The aims of this project are to (1) describe the clinical phenotype of STB (isolated versus disseminated STB), (2) explore different imaging modalities using MRI of the spine and whole-body PET/CT, (3) identify RNA signatures for the two different clinical phenotypes of STB: the isolated and disseminated form of STB, and (4) to analyse the genomes of *Mtb isolates* extracted from different specimens from different sites of the body, hence identifying dual infection. Objectives are listed in Box 1.

**Box 1:** Spinal TB X objectives

Primary objective	To describe the clinical phenotype of spinal TB using whole body PET/CT and to identify mRNA gene expression profiles of isolated spinal TB versus disseminated spinal TB stratified by HIV status
Secondary objectives	1. To identify the distributive patterns of suspected spinal TB using two imaging modalities: MRI and PET/CT 2. To analyse the genomes of *Mtb isolates* from different sites of the body (if available) and to identify differences in their genome regarding SNPs and drug susceptibility 3. To analyse imaging findings using PET/CT at treatment initiation, 6 months, and 12 months to better understand PET/CT as an imaging quality for the judgment of treatment outcome 4. To compare imaging findings on PET/CT and MRI at baseline to evaluate the role of PET/CT in spinal TB diagnostics (specific semi-quantitative PET/CT values as virtual biopsy)

### Study design, setting, and eligibility

This is a prospective cohort study of HIV-infected and -uninfected patients with STB who live in the Cape Town metropole, South Africa. Patients with suspected STB will be referred from Groote Schuur Hospital and its affiliated teaching hospitals (Mitchell`s Plain Hospital, New Somerset Hospital, Victoria Hospital Wynberg) to the study team at the University of Cape Town. Eligibility criteria are listed in Box 2. We aim to recruit 100 participants over a timeframe of three years.

**Box 2:** Eligibility criteria

Inclusion criteria	1. Participant has completed the written informed consent process prior to undergoing any clinical evaluations and willing to undergo HIV testing 2. Spinal TB based on clinical and radiological criteria (TB specific clinical signs and imaging appearance) 3. Age 18 or older with a body weight of at least 40 kg body weight 4. Able and willing to return to follow-up 5. Willing to have samples, including DNA and RNA extraction, stored 6. Willing to consistently practice a highly reliable method of pregnancy prevention
Exclusion criteria	1. Pregnancy or active desire to become pregnant within the next 6 months 2. Uncontrolled diabetes (HbA1c ≥ 6.5% / random glucose concentration ≥ 11.1 mmol/l, fasting plasma glucose ≥ 7.0 mmol/l) 3. Alcohol and substance abuse which might interfere with medication adherence during the trial 4. Positive SARS-CoV-2 PCR in the past 4 weeks 5. Suspicion of malignancy on MRI or known malignancy 6. Suspicion of inflammatory disease and other rheumatological conditions 7. Any person for whom the physician feels this study is not appropriate (e.g., patients already on TB treatment)

### Patient and public involvement

Patients or the public are not involved in the design, or conduct, or reporting, or dissemination plans of this prospective cohort study.

### Case definition

**Fig. 4:**
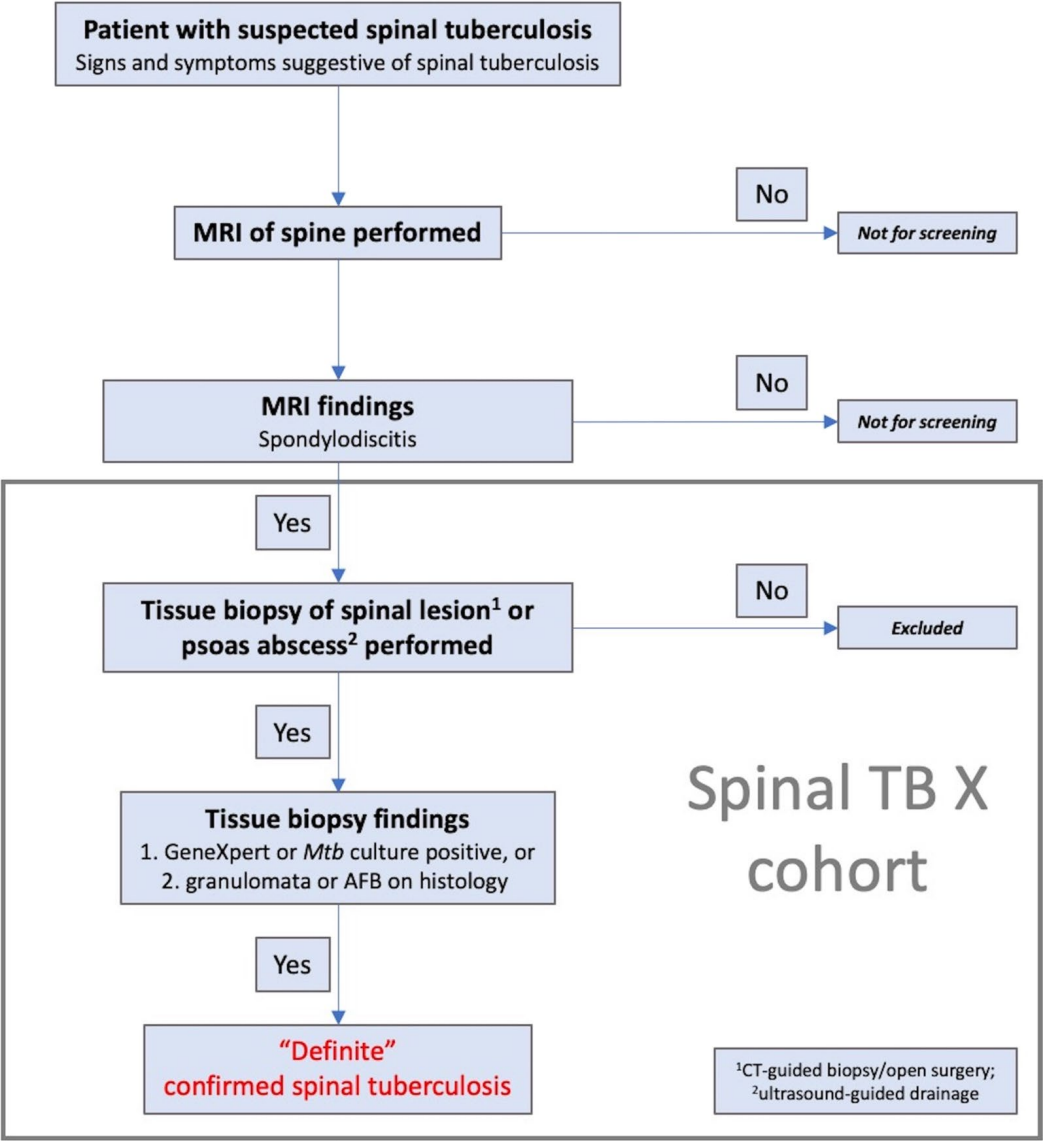
Case definition for "definite" spinal tuberculosis (TB) in the Spinal TB X cohort. *MRI* magnetic resonance imaging, *Mtb* Mycobacterium tuberculosis, *AFB* acid-fast bacilli (Ziehl–Neelsen or auramine-rhodamine stain), *CT* computed tomography

Only cases with clinical signs and symptoms and suspicion of STB on MRI will be included in the Spinal TB X cohort study (Fig. 4). Definite STB is defined as microbiological evidence of *Mtb* infection in tissue of spinal lesions or psoas abscess drainage.

### Study procedures

**Fig. 5:**
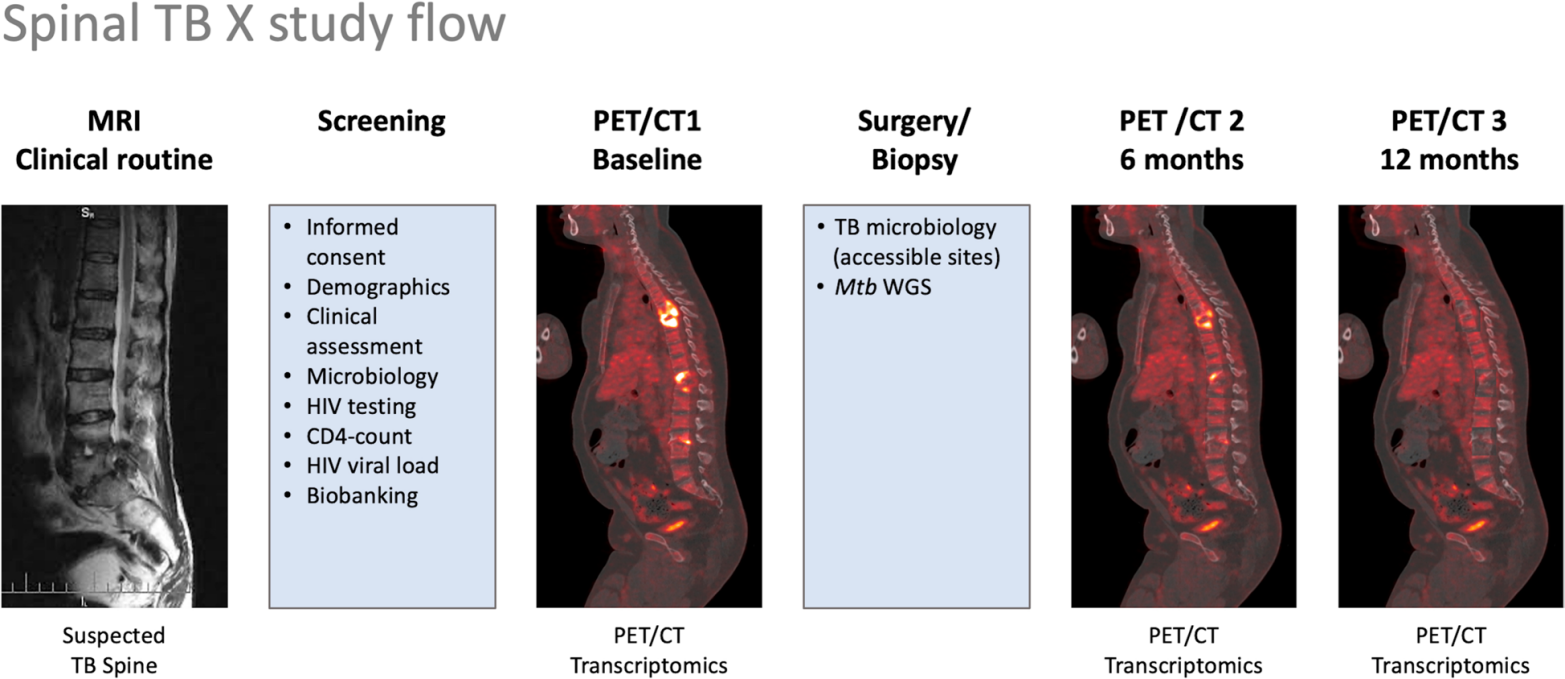
Spinal TB X study flow. *TB* tuberculosis, *MRI* magnetic resonance imaging, *PET*/*CT* ^18^F-fluorodeoxyglucose positron emission tomography/computed tomography

Detailed study procedures and timelines are displayed in Table 1 and Fig. 5. At the pre-screening visit, patients will be assessed regarding their eligibility to join the study. Routine clinical work-up includes blood tests, MRI and work-up for tuberculosis. Patients with suspected spinal tuberculosis on MRI will be approached to join the Spinal TB X cohort study. Informed consent will be obtained by study nurses in the native language of the patient. After inclusion in the study, the screening visit will be performed. At baseline, clinical examination as well as blood collection [creatinine, Alanine transaminase (ALT), full blood count (FBC) including differential count (DIFF), C-reactive protein (CRP) and Haemoglobin A1C (HbA1c)] will be performed. CD4-count, and HIV viral load will be tested in PLWH. Every patient with no history of HIV infection will undergo HIV testing according to national guidelines. Sputum and urine will be collected for GeneXpert Ultra test and *Mtb* culture including drug sensitivity testing, and screening for diabetes and pregnancy will be completed. Whole-body PET/CTs will be performed within ten days after the screening visit. At the PET/CT visit, serum, heparin blood (for Peripheral Blood Mononuclear Cells) and whole blood (in PAXgene tubes) will be collected for biobanking. After the PET/CT, patients will undergo spinal biopsies, or surgery where applicable and specimen will be collected for GeneXpert Ultra test and *Mtb* culture including drug sensitivity testing. Within ten days of the PET/CT visit, patients will be reviewed and examined by the study team and PET/CT findings will be discussed. Where applicable, patients will be referred to medical specialties for further biopsies of sites of infection detected by PET/CT. Patients with microbiologically confirmed "definite" STB on the collected specimen will be started on TB treatment according to local guidelines. Patients with other diagnoses confirmed by culture or histology will be excluded from the study. In the following one to five months, every patient will be contacted by the study team on a regular basis and undergo a standardized questionnaire regarding their clinical course of disease and tuberculosis and HIV (if applicable) drug adherence monitoring. Six months after the first PET/CT, patients will undergo the second PET/CT. At the second PET/CT visit, serum, heparin blood (for Peripheral Blood Mononuclear Cells) and whole blood (in PAXgene tubes) will be collected for biobanking. Additionally, FBC including DIFF and CRP will be collected to determine inflammatory trends. Furthermore, a random-glucose test and in female participants, a beta-HCG-urine test, will be performed. At months seven to eleven, telephonic follow-up will be continued and at month 12, PET/CT 3 will be performed. At the third PET/CT visit, serum, heparin blood (for Peripheral Blood Mononuclear Cells) and whole blood (in PAXgene tubes) will be collected for biobanking. Additionally, FBC including DIFF and CRP will be collected to determine inflammatory trends. Furthermore, a random-glucose test and in female participants, a beta-HCG-urine test, will be performed.

**Table 1:** Spinal TB X study timelines

Visit	Screening	PET/CT 1	Post-PET/CT	Surgery/CT-guided biopsy	M 1–5	PET/CT 2	M 7–11	PET/CT 3
Time point in relation to screening		1 week	2 weeks		Monthly	6 months	monthly	12 months
Visit window (in days)	N/A	± 10	± 10	N/A	± 10	± 10	± 10	± 10
Study IC plus HIV test consent	X							
Vital signs	X	X	X			X		X
Medical history	X	X	X		X	X	X	X
Co-medication	X	X	X		X	X	X	X
Physical examination	X	X	X			X		X
TB specimen collection								
Sputum TB culture (MGIT)^a^	X							
Sputum GeneXpert Ultra	X							
Urine TB culture (MGIT)^a^	X							
Urine GeneXpert Ultra	X							
Site(s) of disease TB culture (MGIT)^a^			X					
Sites(s) of disease GeneXpert Ultra			X					
Spinal tissue TB culture (MGIT)^a^				X				
Spinal tissue GeneXpert Ultra				X				
Blood collection								
Serum chemistry^b^	X					X		X
Full blood count	X					X		X
HBA1C	X							
HIV-1 testing^c^	X							
CD4 + count^d^	X							
HIV-1 viral load^d^	X							
Biobanking (PAXgene, serum, heparin)		X				X		X
Max. blood volume per visit	50 ml	50 ml				50 ml		50 ml
Finger prick								
Glucose	X	X				X		X
Urine collection								
Urine beta-HCG^e^	X	X				X		X
Imaging								
^18^FDG-PET/CT		X				X		X
Follow-up								
Telephonic follow-up					X		X	
Adherence monitoring^f^					X	X	X	X

*PET*/*CT* ^18^F-fluorodeoxyglucose positron emission tomography/computed tomography, *IC* informed consent, *MGIT* Mycobacteria growth indicator tube, *HBA1C* glycated haemoglobin, *CD4* cluster of differentiation, *HCG* Human chorionic gonadotropin

^a^Storage of *Mtb* culture isolates for whole genome sequencing

^b^Alanine transaminase (ALT), creatinine, c reactive protein (CRP)

^c^Participants not on antiretroviral therapy

^d^People living with human-immunodeficiency virus (HIV)

^e^Women only

^f^Tuberculosis and HIV (if applicable) drug adherence questionnaire

### Data collection, data cleaning, and statistical analysis

All study source data will be collected on paper case report forms and entered and stored in RedCap® on a dedicated secure central database at the University of Cape Town. Data will be reviewed by at least two investigators (JS, FT, SM, KW) for completeness and data cleaning, validation and analysis performed by the academic study team. All data will be transferred to R, Version 4.2.2 for all analysis. Normally distributed continuous data will be presented as mean ± SD and non-Gaussian distributed variables as median + IQR. Categorical data will be presented as percentages with 95% CI where appropriate. For patient group comparisons, we will use χ^2^ analysis with calculation of ORs and 95% CI (where appropriate) for discrete variables and student t-test and analysis of variance for normally distributed continuous variables. Multiple logistic regression analyses (entry model) will be performed on age, sex, and baseline characteristics where applicable. Significance will be accepted at the two-sided level of 0.05. Whole-genome and RNA-sequencing will be performed through third parties. Measurement of semi-quantitative PET/CT values will be performed using MIM® Version 7.2.8 (MIM Software Inc., Cleveland, Ohio, USA) according to a standardized reading protocol which allows to compare values between patients with isolated and disseminated STB. Patients which underwent the initial PET/CT but had no confirmed STB diagnosis will serve as control group for the gene-expression profile analysis.

## Discussion

The purpose of this study is to describe the clinical phenotype of spinal TB using whole body PET/CT and to identify a mRNA gene expression profile of isolated spinal TB versus disseminated spinal TB stratified by HIV status. Due to the long follow-up period of the participants, loss to follow-up is anticipated in some of the patients. To counteract this, participants can agree to house visits for such case in the informed consent and provide next of kin details. Further, we follow up participants monthly with telephone calls to keep close contact to the participants and to be aware of any other issues. Participants with significant incidental findings on PET/CT that require immediate diagnostic procedures or treatment may be withdrawn from the study if the investigator deems that continuing participation may not be in the participant’s best interests. At each contact with the participant, information regarding adverse events (AE) will be elicited by appropriate questioning and examinations. All events, both expected/unexpected and related/unrelated will be recorded on a source document. Source documents will include progress notes, laboratory reports, consult notes, phone call summaries, survey tools, and data collection tools. Source documents will be reviewed in a timely manner by the research team. All reportable adverse events that are identified will be recorded on the appropriate case report form (CRF) and in the study chart. The start date, stop date, severity of each reportable event, and the investigator’s judgment of the AE’s relationship and expectedness to the study will also be recorded on the CRF. In the event of a participants` withdrawal from the study due to an AE, it must be recorded on the CRF as such. Adverse events associated with standard of care (TB treatment, biopsies, surgery) will be reported to the Human Research Ethics Committee and marked as study unrelated.

If successful, new diagnostic modalities and treatment plans can be developed, enhancing personalized medicine. Persons with previously undiagnosed medical, surgical, or other conditions identified at screening, including but not limited to PET/CT imaging, HIV infection diagnosis, will benefit from early diagnosis, referral, and rapid access to treatment systems. Similarly, participants who develop new conditions during follow-up will also benefit from early diagnosis and linkage to care. In addition, spinal TB patients will be monitored closely by the study team throughout the study period.

### Supplementary Information

Supplementary file 1

Supplementary file 2

## Data Availability

Not applicable.

## Abbreviations

AE: Adverse event
TB: Tuberculosis
Mtb: Mycobacterium tuberculosis
EPTB: Extrapulmonary tuberculosis
STB: Spinal tuberculosis
MRI: Magnetic resonance imaging
PET/CT: 2-Deoxy-2-[18F]fluoro-d-glucose positron emission tomography/computed tomography
CRF: Case report form
