# Supplementary material for: Comparing gene expression profiles of adults with isolated spinal tuberculosis to disseminated spinal tuberculosis identified by 18FDG-PET/CT at time of diagnosis, 6- and 12-months follow-up: classifying clinical stages of spinal tuberculosis and monitoring treatment response (Spinal TB X cohort study)
Source: J Orthop Surg Res. 2024 Jun 25;19:376. doi: 10.1186/s13018-024-04840-7 (PMC11202394; doi:10.1186/s13018-024-04840-7)
Supplement: Supplementary file 1 [file 13018_2024_4840_MOESM1_ESM.pdf]

Prof. Michael Held, MD, PhD
Department of Surgery, Division of Orthopaedic Surgery, University of Cape Town
michael.held@uct.ac.za

Prof. Friedrich Thienemann, MD, MScIH, DTMPH
Department of Medicine, University of Cape Town
friedrich.thienemann@uct.ac.za

UNIVERSITY OF CAPE TOWN

MAIN STUDY Informed Consent
Protocol no.: GMGHT003
for the research study titled

Comparing gene expression profiles of adults with isolated spinal TB to disseminated spinal TB identified by 18FDG-PET/CT at time of diagnosis, 6- and 12-months follow-up: classifying clinical stages of tuberculosis and monitoring treatment response (Spinal TB X)

Participant initials: ___________________Participant number: _________________

Principal Investigators Name & Qualifications:
A/Prof Friedrich Thienemann, MD, MScIH, DTMPH
A/Prof Michael Held, MD, PhD

Dear Volunteer,

You have been previously diagnosed with spinal TB (via MRI) and we briefly discussed our study with you. Today we would like to explain our study in more detail and see if you are still interested in joining the study. This consent document may contain words that you do not understand. Please ask the study doctor or the study staff to explain any words or information that you do not clearly understand.

Why is this study being done?

Spinal TB (tuberculosis) is an infectious disease of the spine which is usually found in the lung. The origin and the host response towards this disease is to date only poorly understood. It is commonly accepted that the spinal affection of this disease is originated from a primary disease focus in the lung. Usually, the diagnosis of spinal TB is made using an MRI-scan (magnetic resonance imaging) but new data indicates that ^{18}FDG-PET/CT (2-[^{18}F] fluoro-2-deoxyglucose) Positron Emission Tomography/Computer Tomography) can be superior to MRI in diagnosing tuberculosis.

We think that spinal TB can present as two entities: isolated spinal TB with no additional disease locations on whole body PET/CT and disseminated spinal TB with a spinal lesion plus an additional extraspinal lesion on whole body PET/CT and that PET/CT is superior to MRI. Furthermore, we believe that there is a specific gene expression profile which can discriminate between the two entities above only using a finger prick (blood test).

To test this, we would like to take a special picture (whole-body PET/CT) directly after the diagnosis of spinal TB as well as at 6- and 12-months during treatment. Furthermore, at the same time, we would like to take blood samples as well as samples of affected areas (showing in PET/CTs explained above). The entire study period would be 12 months.

Depending on your HIV-test result you will be placed in one of two cohorts.
(Note: both cohorts will receive the same examinations, blood test, radiological test and sample tests).

Whether or not you agree to join this research study, you should know that it is completely up to you and will not affect any medical treatment that you would otherwise receive.

Who is eligible for this study and how many people will be enrolled?

You may be eligible for this study if you:

- If you have MRI confirmed spinal TB
- If you are at least 18 years and weight more than 40 kilograms
- If you are willing and able to return to follow-ups
- If you are willing to have DNA and RNA samples stored
- If you are willing to have blood samples stored
- If you are willing to have other samples (sputum, lymph node, bone marrow) stored
- If you are willing to consistently practice a reliable method of contraception

You will also be tested for HIV for this study. If you do not agree to have an HIV test you cannot be in this study. (There will be more information about HIV testing below.) Since you will be getting radiology scans, we need to make sure you are not pregnant and do not get pregnant while you are on the study. You must agree to use a good method of pregnancy prevention (such as a condom), in addition to any hormonal contraception you may already be on, while you are on the study. It is important to know that the use of condoms even if used correctly does not guarantee that you will not fall pregnant but significantly reduces the risk.

You will NOT be eligible for the study if you:

- If you are pregnant or have the active desire to become pregnant
- If you have uncontrolled diabetes
- If you are consuming uncontrolled amounts of alcohol or other substances which might interfere with medication adherence during the trial
- If you have a positive SARS-CoV-2 PCR in the past 4 weeks
- If you have a suspicion of or have cancer
- If you are a patient for whom the physician feels this study is not appropriate
- If you are younger than 18 years

You also may not be allowed to join the study if you took any experimental medication within the last 3 months, are currently taking certain medicines, or have certain inflammatory diseases.

We plan to include about 100 people in this research study.

HIV testing
Before we can decide in which cohort we can include you in this research study, we must test you for the human immunodeficiency virus (HIV). You cannot participate in this study if you choose not to be tested for HIV. You will receive pre-and post-test counselling. The counselling is conducted by a professional, in a private setting and is strictly confidential

Genetic testing
Some of the blood drawn from you a spart of the study may be used for genetic tests. During genetic testing, we will be examining parts of your deoxyribonucleic acid (DNA) as well as ribonucleic acid (RNA). DNA and the resulting RNA is inherited from your parents and may determine characteristic like e.g., your eye colour. People may have differences in the way they react to the TB infection. Some of these differences may be due to each person`s unique genes. We are asking permission to do tests on your blood to look at the genes that control protection against or susceptibility to spinal TB. This is not the type of testing that allows us to look at your full genetic make-up or that looks at illnesses that are the result of genetic abnormality. We will not be able to give out the results of these special type of tests.

Specimen testing
Depending on the results of your radiological test, you may have more than one site of disease (spinal TB plus extraspinal TB). To analyse the samples of site of disease for testing of differences between the different bacteria sites, we would need to obtain the samples. For example, if we find that you have TB in your lungs, we would like to take sputum. If we find that you have TB in a lymph node, we would like to take a biopsy of this lymph node. If we find that you have TB in your bladder, we would like to obtain some of your urine.
If the treating orthopaedic surgeons feel the necessity for operative treatment, we would like to obtain the samples which are obtained in this procedure.

PET/CT Scanning
A PET/CT scan is a special type of test that helps doctors diagnose diseases like cancer or some infections. The PET/CT scan combines 2 everyday medical imaging machines: positron emission tomography (PET) and computed tomography (CT). Before the scan you are injected with FDG, a type of sugar that gives off a small amount of radiation. The PET scanner can detect and map out the areas in your body that take up the FDG because they are more active than other areas. The CT part of the scan uses x-rays to take pictures of tissues and organs inside the body. The PET and CT images together give a good picture of the structure and activity of your body. The PET scanner is at another location, and we will arrange to transport you there and bring you back. This visit will take most of the day.
Before the test, you will not be allowed to eat (including peppermints, sugar containing medicine or chewing gum) or drink anything but water for about 6 hours. A finger prick will be taken before the PET scan to check your blood sugar level. If your blood sugar is very high, we will delay the scan for about one hour and do another finger prick. If your blood sugar is still high, you will not be able to stay on the study. A pregnancy test, if applicable, will also be done before the scan.
If possible, you should drink 2-3 glasses of water before the test. The FDG will be injected into your vein, and you will rest quietly in a room for about 1 hour, after which the PET scan will be performed. Just before this scan, you will be asked to empty your bladder.

The PET/CT camera is shaped like a big doughnut. You will be asked to lie very still on a table within the machine with your head on a soft cradle and your hands over your head. We will obtain PET images of your body for about 20 minutes, during which time you will hear buzzing and clicking sounds. This scan will be performed with a CT scan. If for any reason you feel that you cannot continue, the scanning can be stopped, and you can be removed from the scanner immediately. However, the information from the scan may be lost at that

time. After the scan is finished, you will be asked to urinate. You should also urinate as often as possible for the rest of the day to help eliminate the radioactive FDG from your body. During the study, we will ask you to have 3 PET/CT scans (in the beginning of the study, after 6 months and after 12 months). A trained radiologist will look at your scans and will tell your doctor if the scan shows something unexpected. The research team will also look at your scans for the study.

If I am eligible and decide to join the study, what will be done to me?

After enrolment, the first step will be the screening. We will examine you (including medical history and medication), obtain sputum samples (sometimes induced sputum will be necessary). We will draw blood for general blood and diabetes tests. Furthermore, we will do HIV-testing as well as viral load tests. We will draw a maximum of 50 ml (10 teaspoons) blood at every visit. To ensure that you are fit for the radiological tests, we will collect urine to determine if you are pregnant (female participants only). We would like to know, that if the samples of the tests performed at every visit are found to be contaminated or are otherwise unevaluable, you will be called back to provide another sample.

If you found to be fit for the study, a PET/CT will be performed within 7 days (+/- 7 days) of screening.

The following will then be performed/obtained at this visit:

1. Examination
2. Blood draw for genetic testing
3. Finger prick for sugar testing
4. If you are at risk of urinary incontinence, a catheter will be placed inside your bladder to avoid any urinary discharge leading to contamination of yourself and others
5. Urine collection for pregnancy testing
6. PET/CT imaging (pregnancy test is negative)

After 7 days after the PET/CT (+/- 7 days) we will do again an examination and will obtain samples of the sites of TB (except spine, which is done in surgery) which were found in the PET/CT.

Between the first and the fifth month, adherence monitoring as well as obtaining your medical history and co-medication will be performed via monthly telephonic follow-ups.

6 months after the first PET/CT, you will undergo a second PET/CT imaging (+/- 7 days). The following will then be performed/obtained at this visit:

1. Examination
2. Blood draw for general blood tests
3. Blood draw for genetic testing
4. Finger prick for random glucose testing
5. In case you are at risk of urinary incontinence, a catheter will be placed inside your bladder to avoid any urinary discharge leading to contamination of yourself and others
6. Urine collection for pregnancy testing
7. PET/CT imaging (pregnancy test is negative)

Between the seventh and the eleventh month, adherence monitoring as well as obtaining your medical history and co-medication will be performed via monthly telephonic follow-ups.

12 months after the first PET/CT, you will undergo a second PET/CT imaging (+/- 7 days). The following will then be performed/obtained at this visit:

1. Examination
2. Blood draw for general blood tests
3. Blood draw for genetic testing
4. Finger prick for random glucose testing
5. In case you are at risk of urinary incontinence, a catheter will be placed inside your bladder to avoid any urinary discharge leading to contamination of yourself and others
6. Urine collection for pregnancy testing
7. PET/CT imaging (pregnancy test is negative)

Each of your study visits will take up to two hours or less. When you have the special scan done, your visit will take a full day.

STUDY SCHEDULE

Spinal TB X - study timelines

Visit	SCR	PET1	POST-PET	*Surgery/CT guided biopsy*	M 1 to 5	PET 2	M 7 to 11	PET 3
**Time point in relation to SCR**		**1 week**	**2 weeks**	***by indication***	**monthly**	**6 months**	**monthly**	**12 months**
Visit window (in days)	N/A	+/- 10	+/- 10	*N/A*	+/-10	+/-10	+/-10	+/-10
**Study informed consent plus HIV test consent**	X							
**Study arm**								
**Vital signs**	X	X	X			X		X
**Medical history**	X	X	X		X	X	X	X
**Co-medication**	X	X	X		X	X	X	X
**Physical examination (including neurology)**	X	X	X			X		X
**TB specimen collection**								
Sputum - TB culture (MGIT) [#]	X							
Sputum - GeneXpert Ultra	X							
Urine- TB culture (MGIT) [#]	X							
Urine - GeneXpert Ultra	X							
Site(s) of disease - TB culture (MGIT)[#]			X					
Sites(s) of disease - GeneXpert Ultra			X					
Spinal biopsy - TB culture (MGIT) [#]				*X*				
Spinal biopsy - GeneXpert Ultra				*X*				
**Blood collection**								
Serum chemistry[1]	X					X		X
Full blood count	X					X		X
HBA1C	X							
HIV-1 testing[2]	X							
CD4+ count[3]	X							
HIV-1 viral load[3]	X							
Blood biomarkers (PaxGene, Heparin, Serum)		X				X		X
Max. blood volume per visit	50 ml	50 ml				50 ml		50 ml
**Finger prick**								
Glucose	X	X				X		X
**Urine collection**								
Urine beta-HCG[4]	X	X				X		X
**Imaging**								
FDG PET/CT		PET1				PET2		PET3
**Follow-up**								
Telephonic follow-up					X		X	
Adherence monitoring[5]					X	X	X	X

[#]Storage of MTB culture colonies at laboratory
[1] ALT, creatinine, hsCRP
[2] For all participants not on ARVs
[3] For participants tested positive for HIV or on ARVs
[4] Women only
[5] TB drug adherence questionnaire

Version 1.0 10 Dec 2021

How long will I be in the study?
You will be on this study for approximately 12 months. As the last visit is an important visit for the study, we encourage you to come back for this visit on time. If you have a problem doing so, we would still like you to come in for a visit at the next possible time you have available. During the study, if you no longer wish to be on the study, or your doctor thinks it is best for you to no longer be on the study, you will be withdrawn from the study.

What are the potential hazards, risks, inconveniences or discomforts?

Taking Sputum

At times, inducing sputum (breathing in the saltwater mist) might be necessary to obtain sputum. This kind of sputum collection may be uncomfortable. Sputum induction may cause wheezing or a tightness in your chest, but it is considered to be a safe procedure.

Drawing Blood
There are minor risks related to drawing blood. You may feel some discomfort when the blood is drawn. You may also have a small bruise or hard bump in your arm where the blood is drawn, and there is an extremely small risk that the skin around the blood draw may get infected. You may also get lightheaded or faint. Hospital or center staff will watch you closely to make sure you get appropriate care if any of these things happen.

Nasal or mouth swab
You may experience some discomfort in the nose when taking the nasal swab and if a mouth swab is taken you may feel like gagging.

Insertion of a urinary bladder catheter
The goal of urinary catheter insertion is to prevent contamination of yourself and the study staff with radioactive substances during the PET/CT visit. You may experience slight discomfort while a small catheter is being inserted into your bladder. In rare cases, the catheter can cause damage to the urethra and / or bladder. Furthermore, in patients with longstanding catheters, there is a risk of urinary tract infections. Very rarely, after removal of the catheter, patients have difficulties voiding.

Risk of Genetic testing
Following genetic testing, your sequence data will be shared in a public database so other investigators may also learn from it (for example, the Database of Genotypes and Phenotypes, dbGaP). Your genotype is your collection of genes. The expression of your genotype contributes to your observable traits, called the phenotype, such as height, eye colour, and blood type. Some traits are largely determined by the genotype, while other traits are largely determined by environmental factors. No personal, identifiable information will be shared in this process, as shared results will be coded with no link back to you. It is possible, however, that someone with a high level of expertise could link anonymous data stored in such a database with an individual person.
Any genetic information collected or discovered about you or your family will be confidential. Records containing this information will be kept on password-protected computer systems and in locked and secured rooms. We will not release any information about you or your family to relatives, any insurance company, employer, or your primary care physician without your written permission.

Biopsies of specimen (e.g., lymph node)
There are minor risks related to taking biopsies. Depending on where in your body the lymph node is, a biopsy can be taken directly (skin prick) or must be done under radiological guidance (CT-Scan, Ultrasound). Usually, the area of pricking will be numbed. You may feel some discomfort when the blood is drawn. You may also have a small bruise or hard bump on the site where the specimen is taken, and there is an extremely small risk that the skin around the blood draw may get infected. You may also get lightheaded or faint. Hospital or center staff will watch you closely to make sure you get appropriate care if any of these things happen.

Radiation Risk
This research study involves exposure to radiation 3 PET/CT scans. This radiation exposure is **not** required for your medical care and is for research purposes only. We measure radiation exposure in mSv. The radiation of three PET/CT scans (43.4 mSv) is less than the maximal permissible annual research exposure of 50 mSv/yr. The average person in the

world receives a radiation exposure of 0.3 rem per year from natural sources, such as the sun, outer space, and the earth's air and soil.
As part of everyday living, everyone is exposed to naturally occurring background radiation. The effective radiation dose from this study is about 15 times higher than background radiation. Roughly 25% of people contract fatal cancer in their lifetime. If you do undergo 3 PET/CT scans during this study (most people will undergo 2), the maximum amount of radiation may increase this risk by roughly 0.3%, to 25.3%. We do not think the amount of radiation in this study will harm you, but we cannot be sure of this. There may be a very slight increase in the risk of cancer.

If you would like more information about radiation, please ask the study doctor.
Please tell your doctor if you have had any radiation exposure in the past year, either from other research studies or from medical tests or care, so we can make sure that you will not receive too much radiation. Radiation exposure includes x-rays taken in radiology departments, cardiac catheterization, and fluoroscopy as well as nuclear medicine scans in which radioactive materials are injected into your body.

If you are pregnant, planning to get pregnant in the next 6 months, or breastfeeding, you will not be permitted to participate in this research study. If you become pregnant, there is risk to the unborn child from the radiation of the scans. It is best to avoid radiation exposure to unborn or breastfeeding infants since they are more sensitive to radiation than adults. You must avoid becoming pregnant while on this study. If you become pregnant or start breastfeeding, we will no longer do PET/CT scans on you.

Other Risks
For your safety, you must tell the study doctor or nurse about all the medications you are taking before you start the study and also before taking any new medications while you are on the study. In addition, you must tell the study doctor or nurse before enrolling in any other clinical trials while you are on this study.

If you are afraid of small spaces and loud noises, you may feel scared when you are having your PET/CT done. This is something you can talk to your doctor about before the scan.

Your participation in the study will be terminated for any of the following reasons:
1) You request to withdraw
2) If your doctor thinks the study is no longer in your best interests
3) Any reason your doctors or study investigators think would justify withdrawal

New findings that develop during the course of this research study that may relate to your willingness to continue participation will be provided to you.

What are the potential benefits to me?

If successful, new diagnostic modalities and treatment plans can be developed and personalized medicine can be enhanced. Persons with previously undiagnosed medical, surgical, or other conditions identified at screening, including but not limited to PET/CT imaging, HIV infection diagnosis, will benefit from early diagnosis, referral and rapid access to treatment systems. Similarly, participants who develop new conditions during follow-up will also benefit from early diagnosis and linkage to care. In addition, spinal TB patients will be monitored closely by the study team throughout the study period.

What other options are there?
Participation in this study is voluntary. Refusal to participate or deciding to stop participation will involve no penalty and will not affect any treatment you are entitled to. If you decide not to participate, any future medical treatment will be the same as that given to other people.

Stored Samples and Future Research
The blood, serum, urine, local specimen, saliva, and sputa samples taken will be stored for research. Samples may be stored at all study sites. We hope that these samples will help us learn more about TB. The research tests we will use are not like regular medical tests. Therefore, we will not put the test results in your medical record; however, if you ask, someone on the study team will discuss the test results with you. If you do not agree to have samples taken and stored for future research, then you cannot take part in this study.

Labelling of Stored Samples
We will label your stored samples with a code that only the study team can link to you. Your information and samples will be stored under a study identification (ID) number and not your name. We will keep any information that can be traced back to you private to the extent permitted by law.

Future Studies
With your consent, we may use your samples for future research. The study team will not send your samples to researchers not listed on the protocol without an ethics committee approval of any new research protocol that requests use of your stored samples. The ethics committee is a committee that oversees medical research studies to protect volunteers' rights and welfare. If samples are sent to outside researchers, the samples will be identified by study ID number only. No names or initials will be included. The study team may also share information such as your sex, age, health history, or ethnicity with outside investigators.

Investigators will use your samples only for research. We will not sell them. Future research that uses your samples may lead to new products, but you will not receive payment for these products. Some future studies may need health information (such as smoking history or present health status) that we don't already have. If so, the study team may contact you for this information.

Risks of stored samples
The greatest risk of allowing us to store your samples will be an unintentional release of your identity from the samples due to release of this information from the stored sample database. Since your name is not in the database, the chances of this happening are very low.

Benefits of stored samples
In general, future research that uses your samples will not help you, but it may help us learn more about tuberculosis.

What about privacy and confidentiality?
All personal information collected as part of this study will be kept strictly confidential, although absolute confidentiality cannot be guaranteed. Any information about you that is linked to your name or other identifying information will be kept in a locked file cabinet that only study staff can access. After the study information about you is collected, it will be put together with information from all other study participants without your name or other identifying information on it. No one other than the study staff, review boards, contracted monitors, and governmental regulatory agencies (South Africa) who review and approve this research study, will be able to link you to the collected information.
When results of a research study are reported in medical journals or scientific meetings, the people who take part are not named or identified and you will be identified only by a code. In most cases, no information will be released about your involvement in the research study without your written permission.

We will keep all information from your medical records private as much as the law allows. The National Health Act, Act No.61, of 2003 in South Africa protects the confidentiality of citizens' medical records. However, you should know that the Act allows release of some information from your medical records without your permission, for example, if it is required by a court order or by law. Part of this National Health Act also requires that certain infectious diseases be reported to the health authorities. One of the diseases that must be reported is TB. The study team will, therefore, make sure that we refer you to your TB clinic if you develop TB again.
A description of this clinical trial will be available on http://www.sanctr.gov.za (as required in South Africa). These Web sites will not include information that can identify you. At most, the Websites will include a summary of the results. You can search these Websites at any time.
Monitors under contract to the sponsor may have access to your research file to monitor all aspects of the study in accordance with the appropriate regulations.
Your medical and research records may be reviewed by the:

- University of Cape Town Faculty of Health Sciences Human Research Ethics Committee
- Local health agencies/authorities (such as the South African Health Products Regulatory Authority)
- The research staff
- The study monitors

By agreeing to participate in this study, you do not waive any rights that you have regarding access to and disclosure of your records. **For further information on those rights, please contact your study doctor Dr Julian Scherer or Prof Michael Held or Prof Friedrich Thienemann whose contact numbers and addresses are shown below.**

Payment, expenses and costs?
Study related costs specified in the protocol will be paid for by the sponsor. Neither you, your medical scheme nor your healthcare provider, will be responsible for these expenses. In other words you will not have to pay for any cost for your participation in this study.
However, if any non-study related health concerns are discovered, and further medical care or counselling are required, you will be responsible for these costs. If you are found to have non-study related health concerns, you will be told where you can get treatment if you do not have your own doctor.
For participating in this study, you will receive R150 for each visit and R500 for each PET/CT scan. If you come on a day other than a study visit for a reason designated by the study staff, you will be compensated R150. This provision has been made to reimburse you for out-or- pocket expenses such as travelling to and from the study site and other miscellaneous costs such as time and inconvenience as a result of study participation.

What are my rights as a participant?
Taking part in this research study is voluntary. You may choose not to take part, or you may leave the study at any time. Leaving the study will not result in any penalty or loss of benefits to which you were otherwise entitled. We will tell you about new information that may affect your health, welfare, or willingness to stay in the study. You have the right to ask any questions you want about this study and about how the information will be used.

Whom do I call if I have any questions or problems?

For the duration of the study, you will be under the care of your study doctor Dr Julian Scherer, Prof Held, Prof Thienemann, and other Sub-Investigators. If at any time you feel that any of your symptoms are causing you any problems, or you have any questions during the study, please do not hesitate to contact the study doctor. If at any time you feel like you are getting sick with TB again, please contact the study team as soon as you can.

Should injury related to the study occur during your participation in this study, immediate medical treatment will be offered to you at no cost. Treatment may involve medications at the study site or referral for hospital admission at the appropriate institution should need be. For questions about the study or a research-related injury, please contact your study doctor **Dr Julian Scherer 076 2030 775**

If you have any ethical concerns or questions and want any information about your rights as a research participant, contact the UCT Faculty of Health Sciences (FHS) Human Research Ethics Committee. The Ethics Committee, and the researchers are responsible for the protection of your rights during this trial. They will answer questions about your rights as a research participant and take any comments or complaints. You can contact Professor Marc Blockman, the Chairman of the UCT FHS Human Research Ethics Committee, by calling 021- 406-6338, or by mail to the following address:

University of Cape Town Faculty of Health Sciences Human Research Ethics Committee
E 53 - Room 46
Old Main Building
Groote Schuur Hospital Observatory
7925

Consent Statement for Study titled: Spinal TB X

☐ I give permission for my sputum, blood, serum, urine, other specimen and saliva samples to be stored indefinitely and used in future research of any type which has been approved by the HREC.

☐ I give permission for my samples to be used for the genetic research described in this consent, or otherwise approved by the HREC.

By signing below, I agree that:
• I have had the chance to ask questions and they were answered to my satisfaction.

• I have been given the time to discuss the information with others and to decide whether or not to take part.

• I will receive a signed and dated copy of this consent form on the day of my signin git.
• I agree to participate in this study.

Printed Name of Participant

____

Participant Signature /Thumbprint Date and Time

____ ___________

Printed Name of Person Conducting Consent
(if other than investigator)

____

Signature and designation of Person Conducting Consent Date and Time
(if other than investigator)

____ ___________

Printed Name of Investigator

____

Signature of Investigator Date and Time

By signing the below, I hereby verify that verbal informed consent was obtained by the above participant. The participant has been informed about the risks and the benefits of the research, understands such risks and benefits and is able to give consent to participation, without coercion, under influence or inappropriate incentives.

*Printed Name of Witness

*Signature of Witness Date and Time
*Where applicable and if thumbprint affixed to consent
