# Supplementary material for: Comparing gene expression profiles of adults with isolated spinal tuberculosis to disseminated spinal tuberculosis identified by 18FDG-PET/CT at time of diagnosis, 6- and 12-months follow-up: classifying clinical stages of spinal tuberculosis and monitoring treatment response (Spinal TB X cohort study)
Source: J Orthop Surg Res. 2024 Jun 25;19:376. doi: 10.1186/s13018-024-04840-7 (PMC11202394; doi:10.1186/s13018-024-04840-7)
Supplement: Supplementary file 2 [file 13018_2024_4840_MOESM2_ESM.pdf]

Study Protocol

Protocol Title

Comparing gene expression profiles of adults with isolated spinal TB to disseminated spinal TB identified by ^{18}FDG-PET/CT at time of diagnosis, 6- and 12-months follow-up: classifying clinical stages of tuberculosis and monitoring treatment response

Short title: Spinal TB X

**Funder**	AO Spine Foundation<br>General Medicine & Global Health (GMGH), University of Cape Town
**Sponsor**	University of Cape Town
**Principal Investigator**	**Friedrich Thienemann, MD, MScIH, DTMPH**<br>General Medicine & Global Health (GMGH), Department of Medicine, and Cape Heart Institute, University of Cape Town<br>Faculty of Health Science, 4th Floor, Chris Barnard Building, Anzio Road, Observatory, 7925 Cape Town, South Africa<br>University Hospital of Zurich and University of Zurich, Rämistrasse 100, 8091 Zurich, Switzerland<br>Phone: +27 21 406 6358<br>Email: friedrich.thienemann@uct.ac.za
**Co-Principal Investigator**	**Michael Held, MD PhD**<br>Department of Surgery, Division of Orthopaedics, University of Cape Town, Observatory, 7925 Cape Town, South Africa<br>Phone: +27 21 404 5118<br>Email: michael.held@uct.ac.za
**Protocol Version & Date**	Version: 2.0 Date: 15 March 2023
**Protocol number**	GMGHT003
**IRB Institute**<br>**IRB Number**	Human Research Ethics Committee, University of Cape Town<br>HREC 243/2022
**Clinicaltrials.gov**	NCT05610098
**Proposed dates**	Start: September 2022 End: December 2025
**Recruitment target**	Screening: 120 Enrolment: 100<br>Follow-up: 12 months
**Investigation**	^{18}FDG-PET/CT
**Location of study**	Cape Town, South Africa

**Co-Investigators**	Julian Scherer, MD, University of Cape Town, South Africa<br>Robert Dunn, MD, University of Cape Town, South Africa<br>Maritz Laubscher, MD, University of Cape Town, South Africa<br>Ferdinand Musawenkosi Oompie, MD, University of Cape Town, South Africa<br>Mariam Qonita Said-Hartley, MD, University of Cape Town, South Africa<br>Hans-Christoph Pape, MD, University of Zurich, Switzerland<br>Karen Wolmarans, MD, University of Cape Town, South Africa<br>Sandra Mukasa, MD, University of Cape Town, South Africa<br>Reto Guler, PhD, University of Cape Town, South Africa<br>Patrick Katoto, MD, University of Cape Town, South Africa<br>Tessa Kotze, University of Cape Town, South Africa<br>Taeksun Song, University of Cape Town, South Africa

CONFIDENTIAL

Protocol chair signature page

Name	Signature	Date
**A/Prof. Friedrich Thienemann**<br>General Medicine & Global Health, Cape Heart Institute, Faculty of Health Sciences, University of Cape Town		17 August 2022
**A/Prof. Michael Held**<br>Department of Surgery, Division of Orthopaedic Surgery, Faculty of Health Sciences, University of Cape Town		17 August 2022
**Dr. Julian Scherer (PhD student)**<br>Department of Surgery, Division of Orthopaedic Surgery, Faculty of Health Sciences, University of Cape Town		17 August 2022

The signatures above constitute the approval of this protocol and the attachments and provide the necessary assurance that this trial will be conducted according to all stipulations of the study protocol, including all statements regarding confidentiality, and according to local legal and regulatory requirements and applicable ICH guidelines.

The study will be carried out in accordance with this protocol, the guidelines and ethical principles set forth in the Declaration of Helsinki, Good Clinical Practice (GCP) and South African GCP. Any proposed changes to this protocol, or to informed consent or participant information documents, will be submitted to the local regulatory authorities for approval prior to implementation.

Table of Contents

Table of Contents 5

Study overview (Synopsis) 7

1 Introduction 9

2 Rationale 9

3 Study Hypothesis and Objectives 10

4 Study design, population, and eligibility 10

4.1 Design 10

4.2 Participant characteristics 11

4.3 Eligibility Criteria 11

4.4 Ethical approval 12

5 Study Schedule and Study Timelines 12

5.1 Participant Screening (SCR) 12

5.2 PET/CT imaging 1 13

5.3 Post PET/CT 13

5.4 Surgery 13

5.5 Month 1 to Month 5 13

5.6 PET/CT imaging 2 13

5.7 Month 7 to Month 11 14

5.8 PET/CT imaging 3 14

6 Detailed description of study procedures 14

6.1 Informed consent 14

6.2 Vital signs & physical examination 15

6.3 Medical History & co-medication 15

6.4 Sputum and urine collection 15

6.5 Blood collection 16

6.6 SARS-CoV-2 testing 17

6.7 Finger prick 17

6.8 Urine beta-hCG 17

6.9 PET/CT scanning 17

6.10 Adherence monitoring 17

6.11 Withdrawal or termination from the study 17

6.12 Study completion 18

6.13 Analysis 18

7 Covid-19 Infection 18

8 Adverse event, serious unanticipated problems, protocol deviations, and non-compliance..18

8.1 Definitions 18

8.2 Investigator Assessment of Adverse Events 19

8.3 Documenting and Recording of Events 21

8.4 Adverse Event Treatment 21

8.5 Expected Adverse Events 21

8.6 Adverse events reporting to IRB 21

8.7 Adverse events reporting to health authorities 21

9 Data handling and record keeping 21

9.1 Confidentiality 22

9.2 Data collection 22

9.3 Data management 22

9.4 Data storage 22

9.5 Data quality management 23

10 Governance 23

11 Potential risks and benefits for the study participants 23

11.1 Potential risks to study participants 23

11.2 Potential benefits to study participants 24

12 Appendices 24

12.1 PET/CT scanners 24

12.2 Remuneration 24

13 References 25

Study overview (Synopsis)

**Title**	Comparing gene expression profiles of adult patients with isolated spinal TB to disseminated spinal TB identified by ^{18}FDG-PET/CT at time of diagnosis, 6- and 12-months follow-up
**Summary**	Tuberculosis (TB) is one of the top ten causes of death worldwide with approximately 10 million cases globally and 1.2 million deaths. Sub-Saharan Africa carries the highest burden of TB. South Africa has one of the highest HIV and TB rates worldwide with an HIV prevalence rate in adults of 19% and a TB case notification rate of 615/100,000 in 2019. Over many years, focus has been paid to pulmonary TB and extrapulmonary TB (EPTB) has received only little attention even though it accounts for almost a quatre of all TB cases. The diagnosis of EPTB remains challenging simply because sample collection requires invasive procedures in the absence of a blood-based diagnostic test. Spinal TB (spondylitis or spondylodiscitis caused by *Mycobacterium tuberculosis*) - often known as Pott`s disease - accounts for up to 10% of EPTB and affects young children, people with HIV-coinfection and elderly, and often leads to lifelong debilitating disease due to devastating deformation of the spine and compression of neural structures. Little is known with regards to the extent of disease and isolated TB spine as well as a disseminated form of TB spine have been described. The latter presents with a spinal manifestation plus disseminations to other organs such as the lungs, pleura, lymph nodes, the GIT or urinary tract or even the central nervous system.<br><br>In our Spinal TB X cohort, we aim to describe the clinical phenotype of spinal TB using whole body PET/CT and identify a specific gene expression profile for the different stages of dissemination and compare our findings to previously described signatures for latent and active pulmonary TB. A blood-based test for spinal TB would lead to earlier diagnosis and treatment in all settings globally and improve treatment outcome of this devastating disease.
**Hypothesis**	1. We hypothesize that spinal TB may present as two clinical phenotypes: isolated spinal TB with no additional lesion on whole body PET/CT and disseminated spinal TB with a spinal lesion plus an additional extraspinal lesion on whole body PET/CT.<br><br>2. We hypothesize that a specific gene expression profile may distinguish between the two clinical entities above and allows for treatment monitoring during antimicrobial therapy ("personalized medicine")<br><br>3. We hypothesize that whole body PET/CT is superior to MRI in diagnosing spinal and extraspinal TB.
**Objectives**	**Primary objective**<br><br>To describe the clinical phenotype of spinal TB using whole body PET/CT and to identify mRNA gene expression profiles of isolated spinal TB versus disseminated spinal TB stratified by HIV status.<br><br>**Secondary objectives**<br><br>1. To identify the distributive patterns of suspected spinal TB using two imaging modalities: MRI and PET/CT.<br>2. To analyse the genomes of *Mtb.* extracted from different sites of the body (if available) and to identify differences in their genome regarding SNPs and drug susceptibility.<br>3. To analyse imaging findings using PET/CT at treatment initiation, 6 months, and 12 months to better understand treatment outcome using PET/CT.<br>4. To compare imaging findings on PET/CT and MRI at baseline to evaluate the role of PET/CT in spinal TB diagnostics (virtual biopsy).

**Design**	This is a prospective cohort study to develop new diagnostic biomarkers for isolated spinal TB versus disseminated spinal TB and treatment monitoring. Furthermore, this study investigates Mtb. strain variations in spinal TB in relation to its distributional pattern. After MRI-confirmation of spinal TB according to local algorithms, patients will be invited to join the study. At baseline, clinical examination as well as blood biobanking will be performed. Every patient with unknown HIV status will undergo HIV-testing. Sputum will be collected for Mtb. culture and GeneXpert as well as specimens from other sites of TB disease (e.g. urine, FNA) and screening for diabetes and pregnancy will be performed. Whole-body PET/CTs will be performed at time of diagnosis (0 month), 6 months, and 12 months of treatment. Either spinal biopsies or surgery will be performed according to clinical indication to confirm the diagnosis of spinal TB at baseline after imaging modalities have been performed (MRI and PET/CT).
**Population**	Patients with newly diagnosed spinal TB at Groote Schuur Hospital and its affiliated hospitals, UCT Private Academic Hospital and other private hospitals in the region.
**Participant Duration**	Approximately 12 months
**Time to complete enrolment**	Approximately 2 years

1 Introduction

In 2019, an estimated 10 million people, out of 1.7 billion carriers of *Mycobacterium tuberculosis (Mtb)*, fell ill with tuberculosis (TB) and 7.1 million patients received TB treatment. Adolescent males accounted for most TB cases in 2019 (56%). 8.2 % of all cases had a positive human immune-deficiency virus-1 (HIV) status. Globally, the highest incidence of TB can be found in South-East Asia (44%) followed by Africa (25%) and the Western Pacific (18%). [1] TB and HIV are two of the leading infectious causes of death worldwide and TB is the leading cause of death in people living with HIV (PLWH). [2, 3] There were approximately 37.9 million PLWH worldwide in 2018, of which almost two thirds (25.7 million) lived in Africa. In South Africa, the country having the largest HIV epidemic in the world, the prevalence of HIV in adolescence (15 to 49 years) was 19% in 2019, whereas the total incidence of TB was 615 per 100,000 in 2019, which leads to the highest burden of HIV co-infected cases globally. [1, 5, 6] The mortality in TB patients almost doubled with HIV co-infections from 22,000 cases (TB without HIV) to 36,000 cases (TB with HIV) in 2019. 7.1% of the previously treated TB cases and 3.4% of the new TB cases in 2019 were resistant to rifampicin and/or Isoniazid in South Africa. (1) PLWH are more likely to develop extrapulmonary TB (EPTB). [11, 12]

Approximately 10-20% of EPTB accounts for skeletal TB with spinal involvement in 50-60% of the cases. [15-17] It is believed that spinal involvement occurs from the spread of *Mtb* from a primary lesion, either pulmonary lesion or from a urogenital infection.

Spinal TB (spondylitis or spondylodiscitis caused by *Mtb*), also called Pott's disease, usually results in local pain, neurological deficit, spinal instability and fever and duration from initial symptoms to adequate diagnosis can take up to several years. [19-21] Diagnosis depends on clinical appearance, imaging, and microbiological confirmation from biopsies. [17] Treatment usually consists of primary chemotherapy, immobilization, and spinal surgery in selected patients. [22] Magnetic resonance imaging (MRI) has a reported specificity of 93% and a sensitivity of 94% in detecting spondylodiscitis and therefore is the current gold standard imaging modality of spinal TB. [23] Spinal cord compression, intrinsic changes of the cord, bony changes of the vertebral body as well as the extent of disc destruction can be detected with high accuracy. [24] If possible, MRI of the whole spine should be performed to detect non-continuous lesions, which occur in 15 to 20% of the patients. [25]

18F-fluorodeoxyglucose (18-FDG) positron emission tomography (PET)/computed tomography (CT) - PET/CT - has recently been shown to be able to detect sites of infection and monitor treatment success. 18-FDG accumulates in tissues with altered glucose metabolism (e.g., malignant conditions, ischemia, inflammatory conditions). It has been found that 18-FDG uptake values are higher in patients with spinal TB compared to patients with pyogenic spondylitis. [43] A recent study has shown that PET/CT is superior to MRI in terms of specificity in the diagnosis of spinal TB. [26] Host transcriptomics have identified a RNA signatures for diagnostics as well as understanding the immunological mechanisms of TB. [27] Several RNA signatures for capillary blood-based PCR-tests have been identified for risk of disease stratification, screening for TB, tracking treatment response and prediction of treatment failure. [28-31] Lately, an RNA signature which predicts recent exposure to *Mtb* in humans has been identified. [32] The description of these signatures are opening the possibility for developing large-scale point of care screening test as well as targeted (personalized) intervention to prevent the disease and, in patients with active tuberculosis, create customized treatment plans. [29]

2 Rationale

The purpose of this proposal is to describe the clinical phenotype of spinal TB using whole body PET/CT and to identify a mRNA gene expression profile of isolated spinal TB versus disseminated spinal TB stratified by HIV status.

Mtb infection of the spine is believed to be due to hematogenous dissemination from a primary focus into the well perfused cancellous bone of the vertebral bodies. [34, 35] With this, the question arises, if isolated TB spine without any other active focus can be a stand-alone entity and if the genome of mycobacterium tuberculosis differs between the different sites of disease. In up to 68% of HIV patients with spinal involvement, mycobacterium tuberculosis was identified as the source of spinal lesions and most of these patients had an advanced HIV-infection, which makes it necessary to stratify this cohort by HIV-status. [39, 40] PET/CT-Scan has recently been shown to be able to detect sites of active infection and monitor treatment response. Additionally, PET/CT may discriminate spinal infection from other spinal processes, and it has been shown that the SUVmax levels in tuberculous

spondylodiscitis are significantly higher compared to cases with pyogenic spondylitis. [42, 43]. In addition, SUVmax will direct to the site for biopsy. [41] Whole-genome sequencing allows for strain typing and identification of multiple strain infection. [44] Different strains have been identified as the cause for a different phenotypical pattern of disease. [45] In our Spinal TB X cohort, we aim to describe the clinical phenotype of spinal TB using whole body PET/CT and identify a specific gene expression profile for the different stages of dissemination and compare our findings to previously described signatures for latent and active pulmonary TB. Currently, the definite diagnosis of spinal tuberculosis is made either by open surgery or CT-guided biopsy to collect specimens for microbiological work up. A blood-based test for spinal TB would lead to earlier diagnosis and treatment in all settings globally and improve treatment outcome of this devastating disease.

3 Study Hypothesis and Objectives

Hypothesis

1. We hypothesize that spinal TB may present as two clinical phenotypes: isolated spinal TB with no additional lesion on whole body PET/CT and disseminated spinal TB with a spinal lesion plus an additional extraspinal lesion on whole body PET/CT.

2. We hypothesize that a specific gene expression profile may distinguish between the two clinical entities above and allows for treatment monitoring during antimicrobial therapy ("personalized medicine")

3. We hypothesize that whole body PET/CT is superior to MRI in diagnosing spinal and extraspinal TB.

Primary objective

To describe the clinical phenotype of spinal TB using whole body PET/CT and to identify and mRNA gene expression profiles of isolated spinal TB versus disseminated spinal TB stratified by HIV status.

Secondary objectives

1. To identify the distributive patterns of suspected spinal TB using two imaging modalities: MRI and PET/CT.
2. To analyse the genomes of *Mtb.* extracted from different sites of the body (if available) and to identify differences in their genome regarding SNPs and drug sensibility.
3. To analyse scintigraphic findings using PET/CT at treatment initiation, 6 months, and 12 months to better understand treatment outcome using PET/CT.
4. To compare imaging findings on PET/CT and MRI at baseline to evaluate the role of PET/CT in spinal TB diagnostics (virtual biopsy).

4 Study design, population, and eligibility

4.1 Design

This is a prospective cohort study to develop new diagnostic biomarkers for isolated spinal TB versus disseminated spinal TB and treatment monitoring. Furthermore, this study investigates genetic variability of Mtb. in spinal TB and its distributional pattern. After MRI-confirmation of spinal TB according to local algorithms, patients will be invited to join the study. At baseline, clinical examination as well as blood biobanking will be performed. Every patient with unknown HIV status will undergo HIV-testing. Sputum will be collected for Mtb. culture and GeneXpert as well as specimens from other sites of TB diseases (e.g. urine, FNA) and screening for diabetes and pregnancy will be performed. Whole-body PET/CTs will be performed at time of diagnosis (0 month), 6 months, and 12 months of treatment. Either spinal biopsies or surgery will be performed according to clinical indication to confirm the diagnosis of spinal TB at baseline after imaging modalities have been performed (MRI and PET/CT).

4.2 Participant characteristics

Participants with clinically and MR-suspected spinal TB will be recruited from Groote Schuur Hospital, Cape Town, South Africa, its affiliated hospitals, UCT Private Academic Hospital and other private hospitals in the region. According to the current caseload, we will be able to include 50 participants per year, aiming for 100 participants in total. However, if recruitment goals cannot be achieved according to aim, Tygerberg Hospital, Cape Town, South Africa may be approached to enhance recruitment. In result, we are aiming for 300 PET/CTs (100 initial, 100 at 6 months, 100 at 12 months) and 300 peripheral blood samples for gene expression analysis.

Spinal TB X - Study Flow

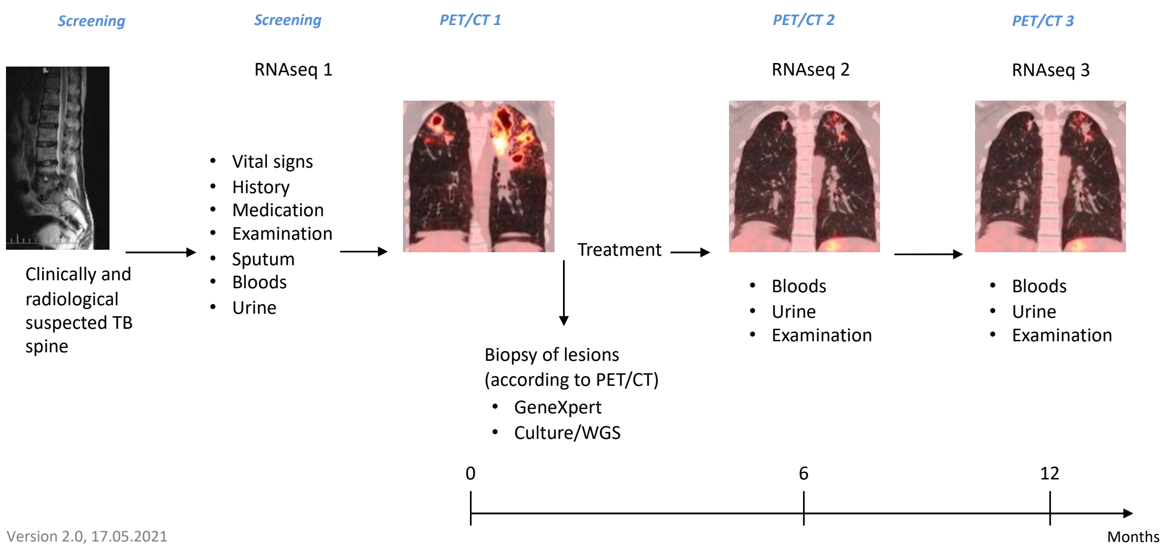

4.3 Eligibility Criteria

Inclusion Criteria

1. Participant has completed the written informed consent process prior to undergoing any clinical evaluations and willing to undergo HIV testing
2. TB spine based on clinical and radiological criteria
3. Age 18 or older with a body weight of at least 40 kg body weight
4. Able and willing to return to follow-up
5. Willing to have samples, including DNA including RNA extraction, stored
6. Willing to consistently practice a highly reliable method of pregnancy prevention

Exclusion Criteria

1. Pregnancy or active desire to become pregnant within the next 6 months.
2. Uncontrolled diabetes (HbA1c ≥ 6.5% / random glucose concentration ≥11.1 mmol/l, fasting plasma glucose ≥ 7.0 mmol/l)
3. Alcohol and substance abuse which might interfere with medication adherence during the trial
4. Positive SARS-CoV-2 PCR in the past 4 weeks
5. Suspicion of malignancy on MRI or known malignancy
6. Suspicion of inflammatory disease and other rheumatological conditions

7. Any person for whom the physician feels this study is not appropriate
8. Age under 18 years

4.4 Ethical approval

The trial will adhere to International Conference on Harmonisation Good Clinical Practice (ICH/GCP) guidelines, and approval will be requested from the University of Cape Town's Faculty of Health Sciences Human Research Ethics Committee (UCT HREC).

5 Study Schedule and Study Timelines

Spinal TB X - study timelines

**Visit**	**SCR**	**PET1**	**POST-PET**	***Surgery/CT guided biopsy***	**M 1 to 5**	**PET 2**	**M 7 to 11**	**PET 3**
**Time point in relation to SCR**		**1 week**	**2 weeks**	***by indication***	**monthly**	**6 months**	**monthly**	**12 months**
Visit window (in days)	N/A	+/- 10	+/- 10	*N/A*	+/-10	+/-10	+/-10	+/-10
**Study informed consent plus HIV test consent**	X							
**Study arm**								
**Vital signs**	X	X	X			X		X
**Medical history**	X	X	X		X	X	X	X
**Co-medication**	X	X	X		X	X	X	X
**Physical examination (including neurology)**	X	X	X			X		X
**TB specimen collection**								
Sputum - TB culture (MGIT) [#]	X							
Sputum - GeneXpert Ultra	X							
Urine- TB culture (MGIT) [#]	X							
Urine - GeneXpert Ultra	X							
Site(s) of disease - TB culture (MGIT)[#]			X					
Sites(s) of disease - GeneXpert Ultra			X					
Spinal biopsy - TB culture (MGIT) [#]				*X*				
Spinal biopsy - GeneXpert Ultra				*X*				
**Blood collection**								
Serum chemistry[1]	X					X		X
Full blood count	X					X		X
HBA1C	X							
HIV-1 testing[2]	X							
CD4+ count[3]	X							
HIV-1 viral load[3]	X							
Blood biomarkers (PaxGene, Heparin, Serum)		X				X		X
Max. blood volume per visit	50 ml	50 ml				50 ml		50 ml
**Finger prick**								
Glucose	X	X				X		X
**Urine collection**								
Urine beta-HCG[4]	X	X				X		X
**Imaging**								
FDG PET/CT		PET1				PET2		PET3
**Follow-up**								
Telephonic follow-up					X		X	
Adherence monitoring[5]					X	X	X	X

[#]Storage of MTB culture colonies at laboratory
[1] ALT, creatinine, hsCRP
[2] For all participants not on ARVs
[3] For participants tested positive for HIV or on ARVs
[4] Women only
[5] TB drug adherence questionnaire

Version 1.0 10 Dec 2021

5.1 Participant Screening (SCR)

Participants with a confirmed diagnosis of spinal TB based on clinical presentation and MRI will be enrolled. participants who sign the **Main Informed Consent Form and HIV-Testing Consent Form** will undergo screening. The following will then be performed/obtained:

1. Vital Signs
2. Medical history and co-medications
3. Physical examination (including neurology)
4. Induced sputum samples (total sputum volume at least 3 mL) for TB culture (MGIT) and GeneXpert Ultra (Xpert).
5. Urine sample for TB culture (MGIT) and GeneXpert Ultra (Xpert)
6. Blood draw for (fasting prior to phlebotomy, max. 50 mL):
    1. Full blood count (including differential), serum chemistry (ALT, creatinine, hsCRP)

2. Diabetes screening (HbA1c)
3. HIV-1 ELISA testing, for all patients not on ART
4. CD4 count and HIV-1 viral load, for all participants tested positive for HIV or on ART

7. Finger prick for random glucose testing
8. Urine collection for pregnancy testing (beta-HCG)

Note that at this and all subsequent visits, if a sputum culture or blood sample is found to be contaminated or is otherwise unevaluable, the participant may be called back to provide another sputum sample. If for some reason a participant needs to come back the next day to provide sputum, this will not be a deviation. In addition, it will not be a protocol deviation if a participant is unable to provide sufficient sputa to perform all sputum-related testing at any study visit.

5.2 PET/CT imaging 1

If eligibility is given, participants will undergo PET/CT imaging within 10 (+/- 10 days) days of screening visit (SCR). The following will then be performed/obtained at this visit:

1. Vital signs
2. Medical history and co-medications
3. Physical examination (including neurology)
4. Blood draw for biomarkers (PaxGene, Heparin, Serum)
5. Finger prick for random glucose testing
6. Urinary bladder catheterization in participants who are (potentially) incontinent
7. Urine collection for pregnancy testing (beta-HCG)
8. PET/CT imaging (pregnancy test is negative)

5.3 Post PET/CT

After PET/CT imaging (within 10 days after PET/CT, +/- 10 days), the following will be performed/obtained at this visit:

1. Vital Signs
2. Medical history and co-medications
3. Physical examination (including neurology)
4. Samples of site(s) of disease (according to PET/CT) if accessible conservatively for TB culture (MGIT) and GeneXpert Ultra (Xpert).

5.4 Surgery

Surgery will be performed by the Department of Orthopaedics if indication is given. Should surgery be performed, spinal biopsies will be obtained for TB culture (MGIT) and GeneXpert Ultra (Xpert).

5.5 Month 1 to Month 5

Between the first and the fifth month, adherence monitoring as well as obtaining participant`s medical history and co-medication will be performed via monthly telephonic follow-ups.

5.6 PET/CT imaging 2

6 months after the first PET/CT, participants will undergo second PET/CT imaging (+/- 10 days). The following will then be performed/obtained at this visit:

1. Vital Signs

2. Medical history and co-medications
3. Physical examination (including neurology)
4. Blood draw for (fasting prior to phlebotomy, max. 50 mL): full blood count (including differential), serum chemistry (ALT, creatinine, hsCRP)
5. Blood draw for biomarkers (PaxGene, Heparin, Serum)
6. Finger prick for random glucose testing
7. Urinary bladder catheterization in participants who are (potentially) incontinent
8. Urine collection for pregnancy testing (beta-HCG)
9. PET/CT imaging (pregnancy test is negative)
10. Adherence monitoring

5.7 Month 7 to Month 11

Between the seventh and the eleventh month, adherence monitoring as well as obtaining participant`s medical history and co-medication will be performed via monthly telephonic follow-ups.

5.8 PET/CT imaging 3

12 months after the first PET/CT, participants will undergo third PET/CT imaging (+/- 10 days). The following will then be performed/obtained at this visit:

1. Vital Signs
2. Medical history and co-medications
3. Physical examination (including neurology)
4. Blood draw for (fasting prior to phlebotomy, max. 50 mL): full blood count (including differential), serum chemistry (ALT, creatinine, hsCRP)
5. Blood draw for biomarkers (PaxGene, Heparin, Serum)
6. Finger prick for random glucose testing
7. Urinary bladder catheterization in participants who are (potentially) incontinent
8. Urine collection for pregnancy testing (beta-HCG)
9. PET/CT imaging (pregnancy test is negative)
10. Adherence monitoring

6 Detailed description of study procedures

6.1 Informed consent

Informed consent is a process where information is presented to enable persons to voluntarily decide whether to participate as a research participant. It is an ongoing conversation between the participant and the researchers, which begins before consent is given and continues until the end of the participant's involvement in the research. Discussions about the research will provide essential information about the study and include purpose, duration, procedures, alternatives, risks, and benefits. Participants will be given the opportunity to ask questions and have them answered.

There will be a **Main Informed Consent Form and HIV-Testing Consent Form**. If the person is potentially eligible and interested in joining the trial, the Main Consent Form and HIV-Testing Consent Form will be used at the participant's screening visit and before any study related procedures are performed. The informed consent process for both Consent Forms will be conducted by research staff such as clinical research workers, nurses, study coordinators, doctors, or other qualified investigators. The person obtaining informed consent will ask questions to assess the participant's understanding of

the mentioned Consent Forms and state that participation is voluntary and that participants may refuse participation or withdraw at any time without prejudice to their clinical care.

The consent forms will be prepared in English and translated to Xhosa and Afrikaans.

The participant will sign the informed consent documents prior to undergoing any procedures. The researcher will also sign the ICF and document the consent process in the participant's research and/or medical record. Participants will also be informed that study results will be made available to them in the future by means of pamphlets, leaflets, phone calls, SMS or radio broadcasting. Consent forms will be stored securely at study sites as per GCP requirements.

6.2 Vital signs & physical examination

Vital signs

Vital signs will be performed at every visit by a nurse or nurse assistant. Vital signs include heart rate, respiratory rate, blood pressure, body temperature, height, and weight.

Physical Examination

A detailed complete physical exam will include examination of lymph nodes, cardiac and respiratory systems, abdomen, skin, back/spine, and neurology. In addition, various assessment scores will also be performed. Examination and assessment will be recorded in a structured manner. A complete physical exam will only be performed at the screening visit (SCR).

A focused physical exam will be directed only at new symptoms and complaints of the participant to find evidence of active disease. This targeted physical exam may include examination of lymph nodes, respiratory system, abdomen and back/spine and neurology. A focused physical exam will be performed at every visit except the screening visit (SCR).

6.3 Medical History & co-medication

Medical History

A complete medical history will be taken and recorded in a structured format only at the screening visit (SCR). The complete medical history will cover current symptoms, past medical history, occupational history, family and social history, drug history, allergies, and review of systems.

A focused medical history will be taken for cause and recorded in a structured format. The focused medical history will cover new symptoms and review of systems, as needed. A focused medical history will be taken at every visit the screening visit (SCR).

Co-medication

Co-medication will be recorded at screening visits in detail (type, dose, route, frequency) and source documents from the primary care clinics will be used for verification. This information will be updated at every follow-up visit throughout the study. All co-medication will be recorded on the co-medication.

6.4 Sputum and urine collection

Sputum induction

Sputum induction at screening will take place in a designated area. Hypertonic saline will be administered via the mouthpiece of a nebulizer to induce expectoration. Staff members assisting participants will wear N95 respiratory protection masks. Sputum induction is not required if the participant can produce sputum without induction.

TB culture (MGIT)

Sputum and urine will be cultured on liquid media (Mycobacteria Growth Indicator Tube, MGIT) at screening visit to determine pulmonary TB status. Contaminated cultures will be considered “no test”, i.e., neither culture positive nor negative, and will be repeated**.** To note, if a culture is contaminated or otherwise not evaluable, another sputum sample may be requested prior to the next study visit. Isolates of positive MGIT cultures will be harvested and stored. This includes also positive Mtb cultures of another site of disease.

GeneXpert Ultra

GeneXpert Ultra will be performed at screening visits to determine pulmonary TB status. GeneXpert Ultra will be used to measure cycle threshold (the amplification cycle when Mtb DNA becomes detectable in the Xpert assay) as a surrogate marker for bacterial load. Gene Xpert Ultra may be done if sputum or sputum sediment is available.

6.5 Blood collection

Approximately 50 mL of blood will be drawn at each time point as indicated on the study timelines. Samples will be processed in the haematology and biochemistry laboratories of the National Health Laboratory Service according to local SOP and the research laboratories. Testing may be repeated to check for or follow-up on abnormal results when needed, for example, at screening or after learning about an adverse event.

Full blood count

Up to 4 mL of blood will be collected in an EDTA tube for haematology (full blood count and differential).

Serum chemistry

Up to 5 mL of blood will be collected in a clot activator tube (with or without gel) for serum collection. The serum can be used for performance of ALT, creatinine and hsCRP.

HBA1C

Up to 4 mL of blood will be collected in an EDTA tube for HBA1C.

HIV-1 testing

Up to 5 mL of blood will be collected in a clot activator tube (with or without gel) for serum collection. The serum will be used for HIV-1 ELISA test for all participants not on ART.

CD4 count

Up to 4 mL of blood will be collected in an EDTA tube for HB

HIV-1 viral load

Up to 3 mL of blood will be collected in an EDTA tube for

Blood biobanking - immunological markers

Up to 4 mL will be collected in an SST tube (serum separator tube) for immunological markers (cytokines, chemokines, soluble mediators).

Blood biobanking – PBMC isolation

Up to 36 mL will be collected for sodium heparin tube for PBMC isolation for macrophage assays, confocal and CFU assays.

Blood biobanking – blood for host mRNA

Approximately 5 mL blood will be collected in two separate PAXgene tubes (2x2.5 mL) for determining host mRNA signatures at three different time points: baseline, 6 months, 12 months.

Analysis will be performed by a third-party company (Inflammatix), any other academic group or the South African MRC.

6.6 SARS-CoV-2 testing

Naso- or oro-pharyngeal swap for SARS-CoV-2 RT-PCR or Rapid Ag testing will be performed if symptomatic or required for the MRI, PET/CT or any other protocol investigation. Serum safe may be sued for SARS-CoV-2 antibody testing.

6.7 Finger prick

A fingerpick will be performed for a test for blood glucose levels.

6.8 Urine beta-hCG

Urine will be collected for pregnancy testing before each PET/CT scan. A commercial human chorionic gonadotropin (beta-hCG) determination assay will be performed in accordance with manufacturers' guidance.

6.9 PET/CT scanning

The whole-body PET/CT scans will be performed at Cape Universities Body Imaging Centre (CUBIC) at the University of Cape Town (UCT). The CT portion of the scan will be done without contrast. Participants will consent to receive a maximum of 3 PET/CT scans during the study (PET 1, PET 2 and PET 3).

Participants will be fully briefed regarding what to expect and any precautions highlighted. Participants will be asked not to eat for about 6 hours prior to the scan but to drink plenty of water. Participants will have an assessment of blood sugar. A venous cannula will be inserted and approximately 7 mCi of radiolabelled Fluorodeoxyglucose (^{18}F) administered. In case of expected incontinence and if the participant is not already catheterized, a urinary catheter will be inserted. After about 50 minutes participants will void urine, and at about 60 minutes after injection, the participants will undergo a whole-body PET/CT. For details of the scanners used, please refer to the appendices.

6.10 Adherence monitoring

Adherence to ART and/or TB-treatment will be monitored by study staff at visits and via telephonic follow-up.

6.11 Withdrawal or termination from the study

Participants with significant incidental findings on PET/CT that require immediate diagnostic procedures or treatment may be withdrawn from the study if in the opinion of the investigator, continuing the study may not be in the participant's best interests. Participants will be replaced.

During any stage of the study, participants may be withdrawn and replaced if:
a) Participant withdraws consent
b) There is any reason deemed appropriate by the investigator or attending physician.

6.12 Study completion

Participation will be considered completed if the participant completes the final (third) PET/CT.

6.13 Analysis

Analysis will be performed using SPSS®. Continuous data will be analysed as median and interquartile range [IQR] and categorical data as frequencies (%) with their 95% confidence interval (CI) where appropriate. For comparing proportions between two groups, we use the chi-square (v2) test. Medians will be compared between two groups (HIV infected and HIV uninfected) using the Wilcoxon ranksum test. Univariate logistic regression will be performed and the cut-off P-value < 0.2 used for inclusion of variables in the multivariate logistic regression model. Multivariate logistic regression will use a stepwise backwards and manual intelligent techniques to identify the independent predictors of the composite endpoint of MS. The logistic regression models' results will be presented as odds ratios (OR) with the 95% CI. Statistical significance will be accepted at the two-sided level of $P \le 0.05$. Sociodemographic and clinical data as well as laboratory findings will be analysed.

Semiquantitative PET/CT analysis will be performed using MIM® Software. Total lesion glycolysis (TLG) at the region of interest (ROI) will be measured as well as SUVmean, SUVmax and SUVvolume after subthreshold calculation of the ROI.

7 Covid-19 Infection

The coronavirus disease 2019 (COVID-19) is a respiratory viral infection caused by the Severe Acute

Respiratory Syndrome Coronavirus 2 (SARS-CoV-2) virus. The SARS-CoV-2 originated in Wuhan, China in December 2019 and was declared a global pandemic by the WHO on March 11, 2020. COVID-19 symptoms include fever, chills, cough, shortness of breath, fatigue, loss of smell and test, myalgias, and headache amongst others. These symptoms may overlap with those of pulmonary tuberculosis. How a coinfection with SARS-CoV-2 affects the clinical presentation or treatment outcome of pulmonary TB patients is not currently well known.

Covid-19-PCR-tests or Antigen-tests will be performed at any visit according to the present local epidemiology and guidelines.

8 Adverse event, serious unanticipated problems, protocol deviations, and non-compliance

8.1 Definitions

Adverse Event (AE)

Any untoward medical occurrence in a participant, including any abnormal sign, symptom, or disease, temporally associated with the participant's participation in research, whether or not considered related to the participant's participation in the research.

Serious adverse event (SAE)

Any adverse event that:

a) results in death.

b) is life-threatening (places the participant at immediate risk of death from the event as it occurred).

c) results in in-patient hospitalization or prolongation of existing hospitalization.

d) results in a persistent or significant disability/incapacity.

e) results in a congenital anomaly/birth defect; or

f) based upon appropriate medical judgment, may jeopardize the participant's health, and may require medical or surgical intervention to prevent one of the other outcomes listed in this definition

Protocol Deviation (PD)

Any change, divergence, or departure from the Institutional Review Board (IRB) approved study procedures in a research protocol. Protocol deviations are designated as serious or non-serious and are further characterized as the following:

a) Those that occur because a member of the research team deviates from the protocol

b) Those that are identified before they occur, but cannot be prevented

c) Those that are discovered after they occur

Serious Protocol Deviation (SPD)

A deviation that meets the definition of a Serious Adverse Event (SAE) or compromises the safety, welfare or rights of participants or others.

Unanticipated Problem (UP)

Any incident, experience, or outcome that meets all three of the following criteria would be considered a serious UP:

1. unexpected in terms of nature, severity, or frequency in relation to
    - the research risks that are described in the IRB-approved research protocol and informed consent document or other study documents; and
    - the characteristics of the participant population being studied
2. related, possibly or probably related to participation in the research
3. suggests that the research places participants or others at a greater risk of harm (including physical, psychological, economic, or social harm) than was previously known or recognized.

Unanticipated problem that is not an AE (UPnonAE)

An unanticipated problem that does not fit the definition of an AE, but which may, in the opinion of the investigator, involve risk to the participant, affect others in the research study, or significantly impact the integrity of research data. These events may involve a greater risk of social or economic harm to participants or others rather than physical/psychological harm. Such events would be considered a non- serious UP. Examples of an UPnonAE include a breach of confidentiality, accidental destruction of study records, or unaccounted-for study drug.

8.2 Investigator Assessment of Adverse Events

Grading Adverse Events for Severity

The severity of each AE will be determined using the corrected version of the Division of AIDS (DAIDS) Table for Grading the Severity of Adult and Pediatric Adverse Events. This table can be found using the following link: DAIDS TOX TABLE (Corrected Version 2.1 July 2017). Any events that are not listed in this toxicity table will be graded by the local investigator as follows:

Table 1: Adverse event grading

Grade 1 - Mild	Transient or mild discomfort; no limitation in activity; no medical intervention/therapy required
Grade 2 - Moderate	Moderate limitation in activity – some assistance may be needed; no or minimal medical intervention/ therapy required
Grade 3 - Severe	Marked limitation in activity; some assistance usually required; medical intervention/therapy required; hospitalizations possible
Grade 4 - Life-threatening	Extreme limitation in activity, significant assistance required; significant medical intervention/therapy required, hospitalization or hospice care probable
Grade 5 - Death	

Assessing Adverse Events (AE) for relationship to study

Any AE that occurs in a participant will be assessed for its relationship to the study. A causal relationship means an intervention caused (or is reasonably likely to have caused) the AE. This usually implies a relationship in time between one or more intervention and the AE - for example, the AE occurred shortly after the participant received the drugs/study agents/intervention.

For all AEs, the clinician who examines and evaluates the participant will determine the AE's causality based upon the temporal relationship to administration of the intervention, the pharmacology of any applicable study agents, and his/her clinical judgment.

The following scale will be used to reflect the PI's judgment as to the relationship between the intervention and the AE:

Definitely Related

The AE is clearly related to one or more of the interventions – follows a reasonable temporal sequence from administration of one or more of the interventions, follows a known or expected response pattern to the one or more of the interventions that is confirmed by improvement on stopping and reappearance of the event in repeated exposure and that could not be reasonably explained by the known characteristics of the participant's clinical state.

Probably Related

The AE and administration of the interventions are reasonably related in time and/or follows a known pattern of response, and the AE is more likely explained by one or more of the interventions than other causes.

Possibly Related

AE follows a reasonable temporal sequence from administration of the interventions, follows a known or expected response pattern to the suspected intervention or interventions, but that could readily have been produced by several other factors.

Unlikely Related

A potential relationship between one or more of the interventions and the AE could exist (i.e., the possibility cannot be excluded), but the AE is most likely explained by causes other than one or more of the interventions (e.g., could readily have been produced by the participant's clinical state or could have been due to environmental or other interventions)

Unrelated

AE is clearly not related to one or more of the interventions - another cause of the event is most plausible and/or a clinically plausible, temporal sequence is consistent with the onset of the event and the intervention administration and/or event is biologically implausible.

8.3 Documenting and Recording of Events

At each contact with the participant, information regarding adverse events will be elicited by appropriate questioning and examinations. All events, both expected/unexpected and related/unrelated will be recorded on a source document. Source documents will include progress notes, laboratory reports, consult notes, phone call summaries, survey tools and data collection tools. Source documents will be reviewed in a timely manner by the research team. All reportable adverse events that are identified will be recorded on the appropriate case report form (CRF) and in the study chart. The start date, stop date, severity of each reportable event, and the investigator's judgment of the AE's relationship and expectedness to the study will also be recorded on the CRF. In the event of a participants` withdrawal from the study due to an AE, it must be recorded on the CRF as such. Adverse events associated with standard of care (TB treatment, biopsies, surgery) will not be reported.

8.4 Adverse Event Treatment

Once an AE is known, staff at the study site should ensure that the participant receives prompt and appropriate care. Should a participant call a study clinician to report an AE, it will be determined at that time if an extra visit(s) will be scheduled, in addition to providing appropriate medical advice. All actions taken by the investigator after observing an AE should be documented, including increased monitoring of the participant, suspension of any treatment, etc. Additionally, all calls will be documented in the participant's study chart.

8.5 Expected Adverse Events

Expected adverse events are listed in Table 2.

Table 2: Expected adverse events

**Intervention/potential cause of AE**	Adverse effects
**Blood drawing**	Common: Discomfort
	Significant but rare: Hematoma, Infection, nerve damage, syncope
**FDG-PET/CT**	Significant but rare: Hematoma, thrombophlebitis, infection, nerve damage, syncope
**Induced Sputum**	Can have coughing, wheezing, and or bronchospasms

8.6 Adverse events reporting to IRB

Adverse events will be reported to UCT Human Research Ethics Committee according to the HREC Standard Operation Procedures (SOP), University of Cape Town, Version 7.0, April 2019.

8.7 Adverse events reporting to health authorities

Adverse events will be reported to South African Health Products Regulatory Authority (SAHPRA) according to SAHPRA SAFETY REPORTING DURING CLINICAL TRIALS IN SOUTH AFRICA, Version 2, August 2019.

9 Data handling and record keeping

9.1 Confidentiality

All study staff, the sponsor, and any sponsor representatives; will preserve the confidentiality of all participants taking part in the study in accordance with ICH GCP, applicable South African national and local regulations and (to the extent applicable) the U.S. Health Insurance Portability and Accountability Act of 1996 (“HIPAA”). Information about trial participants will be kept confidential. All trial data will be de-identified and coded with a study number. Only participant study number and initials will be used on the CRF and in all study correspondence. The trial enrolment log and all signed ICFs and case report forms (CRFs) will be stored in a locked cabinet that is ICH-GCP compliant.

9.2 Data collection

Study data will be collected on standardized paper CRFs. These forms are to be completed on an ongoing basis during the study. Any type of corrections to paper CRFs must be initiated and dated by the person making the correction. The PI is responsible for assuring that the data collected are complete, accurate, and recorded in a timely manner. The CRFs will be collected and placed into a participant- specific binder. Source documentation (the point of initial recording of a piece of data) should support the data collected on the CRF and be signed and dated by the person recording and/or reviewing the data. Some CRFs may also be source documents. Source documents include all recordings of observations or notations of clinical activities and all reports and records necessary for the evaluation and reconstruction of the study. Source documents include, but are not limited to, the participant medical records, electronic chart records, laboratory reports, x-rays, radiologist reports, biopsy reports, ultrasound photographs, participant progress notes, pharmacy records and any other similar reports or records of procedures performed during the participant’s participation in the protocol. Data for CRFs will be collected during participant visits by health care providers and abstracted from the medical record. Once the data is collected, it will be reviewed by the local site monitoring team or their contractors. Any data compiled for statistics or other manipulation will be handled in a redcap database. The scientific results from this study will require various formats, depending on the data type. Locked copies of these files containing the results will be compiled by the principal investigator and made available to monitoring and regulatory agencies as necessary.

9.3 Data management

Data from the CRF will be entered into a data entry system developed and managed by the study team using a double data entry system to assist with accuracy, authenticity, and completeness. Access to the database will be password controlled and will be limited to those with data entry and management responsibilities, as well as monitors. Staff members delegated to capture data will be trained on how to enter data from the paper CRFs onto the database, how to check data prior to data capture and how to clean and validate data. Data capturers must initial and date each paper CRF once captured. Records are protected by ownership control and the database provides an audit trail so that all additions, changes and deletions to the data and the data entry system are tracked. All study related data including imaging data will be maintained on servers located in South Africa or Switzerland.

9.4 Data storage

During the study duration all study participant essential documents will be kept in a securely locked cabinet. When study files are removed from the storage cabinet in the file room, they must be signed out and signed in when they are returned to permit the tracking of files. Data entered in the data entry system will be hosted on UCT servers which are backed up on a daily, weekly, and monthly basis. Backup data can be retrieved on request through the UCT ICTS department. Electronic data will be archived using archive storage space on UCT servers dedicated to archive material and on the UCT institutional data repository service, ZivaHub as per
http://www.digitalservices.lib.uct.ac.za/dls/deposit-guidelines

All essential documentation for all study participants including history and physical findings, laboratory data, and results of consultations are to be maintained by the investigators in a secure storage facility for a minimum of three years. These records are to be maintained in compliance with UCT REC, local and government requirements, whichever is longest. All records are to be kept confidential.

9.5 Data quality management

The PI will supervise study staff and data capturers and ensure that they responsibly manage the data from its recording to its capture in the database ensuring complete, high quality study data.

The variables indicated on the paper CRFs, validated against the trial protocol, will be used to develop the database. During the development stage, data validation tests will be performed on each data entry field to ensure the data entry system provides accurate, valid data sets. Sample data will be entered into each field on every CRF in the database to ensure the field allows entry of valid data and detects all entries that violate the correct format for that field or any constraints for that field. Any errors detected will be fixed and the design re-validated thereafter. Data Capture staff will undergo training on the database before the study starts. Refresher training will take place on a regular basis (3/6 monthly) or as required should problems arise with the data capture process.

Appropriate administrative, physical, and technical safeguards to ensure the confidentiality, integrity and security of electronic health information will be taken. The data entry systems user permission structures provide auditing trails in line with international requirements. Access to the database is password controlled and will be limited to those with data entry and data management responsibilities.

The database will have automated data edit checks and validation procedures applied within a field and across fields in a CRF or multiple CRFs. Data that does not comply with these rules will be highlighted and entry will not be permitted, minimising data capture errors. Data Capturers will log a query on any field in the database where there is uncertainty regarding the accuracy of the data to be entered. The query must be flagged with a 'post-it' note on the hard-copy CRF and confirmation must then be requested from clinical staff members. The clinical staff member will resolve the query, update the CRF where applicable and then update the QC page.

Data quality can be improved by catching errors during data capture from paper CRFs. Data capturers must ensure that the two levels of QC have been completed. In addition, data quality will be improved by a double data entry system. There will be a reconciliation process between the first and second data entry of each CRF. This process will be done by the second entry personnel. All discrepancies between the two entries will be identified and the correct entry will be identified in the data entry system as the final entry for that field. This will be done immediately after the second data entry is completed capturing the data.

The data manager will conduct a review of the data captured in the database, checking for errors, inconsistencies, and missing data. The query log, discrepancy report and the validation reports containing inconsistencies and missing data will be generated on a two-week basis for discussion at the study team meeting.

10 Governance

Governance of the study

The Clinical Study Oversight Committee (CSOC) will monitor study specific issues (e.g., recruitment, safety, regulatory) and will be comprised of Prof Thienemann, Prof Held, Dr Sandra Mukasa, Dr Julian Scherer, and Prof Reto Guler.

The CSOC will have a meeting every two months with conference call link-up with those members not in Cape Town. The CSOC will review preparation for the study, clinical enrolment, and events on the study as well as all other aspects such as analysis and sub-studies. For each meeting of the CSOC a progress report will be prepared that will include participant accrual, study primary endpoint and deaths (blinded to study arm), all serious adverse events with details of each individual serious adverse event. Drs Sandra Mukasa/Julian Scherer and Profs Friedrich Thienemann/Michael Held will be responsible for day-to-day management of the project and for human resources issues.

11 Potential risks and benefits for the study participants

11.1 Potential risks to study participants

There are minor risks related to blood drawing, including discomfort, hematoma, and rarely an infection. Sputum collection may also be uncomfortable and sputum induction can cause wheezing or

a tightness in the airways. It is generally thought to be a safe procedure. Insertion of a urinary catheter can rarely result in a urethral injury, urinary tract infection or urinary retention after exertion. There is a risk, although rare, that placing an intravenous line for the PET/CT may result in a hematoma, thrombophlebitis, infection, or nerve damage. There is also potential risk associated with radiation exposure from PET/CT imaging (**Table 3**). Both PET and CT components of the scan will expose participants to ionizing radiation. Participants will undergo three PET/CT scans during the study period of 12 months. PET/CT scans will be captured from the base of the skull to the sub-trochanteric region.

**Scan**	**PET1 (dose in mSv)**	**PET2 (dose in mSv)**	**PET3 (dose in mSv)**
**^{18}FDG PET**	**4.8**	**4.8**	**4.8**
**CT**	**15 (standard dose)**	**7 (low dose)**	**7 (low dose)**
**Total**	**19.8**	**11.8**	**11.8**

Table 3: Total maximum radiation dose in South Africa.

The above table shows the maximum projected dose per scan. Most participants will be scanned 3 times and will receive about 43.4 mSv. The radiation of three PET/CT scans is less than the maximal permissible annual research exposure of 50 mSv per year [34].

11.2 Potential benefits to study participants

If successful, new diagnostic modalities and treatment plans can be developed and personalized medicine can be enhanced. Persons with previously undiagnosed medical, surgical, or other conditions identified at screening, including but not limited to PET/CT imaging, HIV infection diagnosis, will benefit from early diagnosis, referral, and rapid access to treatment systems. Similarly, participants who develop new conditions during follow-up will also benefit from early diagnosis and linkage to care. In addition, spinal TB patients will be monitored closely by the study team throughout the study period.

12 Appendices

12.1 PET/CT scanners

The PET/CT scans in South Africa will be performed on a Siemens Biograph mCT 64 flow PET/CT. Participants in South Africa will be scanned using the following parameters: 120 kV, 200 mA, 0.75 seconds rotation time, and a pitch of 0.438 with a collimation of 16 X0.75 mm for the 1st, 2nd and 3rd scan. The radiation is as presented in table 3. The CT portion of the PET/CT will be used as an attenuation correction for the PET signal.

12.2 Remuneration

Study participants will receive the following compensation for each type of visit. If possible, the participants will be compensated via SMS.

Table 4: Remuneration/compensation for study visits

Study visit	Remuneration/compensation
Screening	**150**
Each PET/CT	**500**
Post PET/CT 1	**150**
Extra sputum visit or blood draw	**150**

Participants may receive further reimbursements, such as a phone card or food and drinks while waiting.

13 References

1. World Health Organization, *Global tuberculosis report 2020*. 2020, Geneva.
2. Organization, W.H., *Global tuberculosis report 2013*. 2013: World Health Organization.
3. Diedrich, C.R., J. O'Hern, and R.J. Wilkinson, *HIV-1 and the Mycobacterium tuberculosis granuloma: A systematic review and meta-analysis.* Tuberculosis (Edinb), 2016. **98**: p. 62-76.
4. World Health Organization *Fact sheets: HIV/AIDS*. 2019.
5. UNAIDS *HIV/AIDS South Africa*. 2020.
6. Naidoo, P., et al., *The South African Tuberculosis Care Cascade: Estimated Losses and Methodological Challenges.* J Infect Dis, 2017. **216**(suppl_7): p. S702-S713.
7. Kwan, C.K. and J.D. Ernst, *HIV and tuberculosis: a deadly human syndemic.* Clin Microbiol Rev, 2011. **24**(2): p. 351-76.
8. Barnes, P.F., et al., *Tuberculosis in patients with human immunodeficiency virus infection.* N Engl J Med, 1991. **324**(23): p. 1644-50.
9. Bender, B.S., et al., *Role of the mononuclear phagocyte system in the immunopathogenesis of human immunodeficiency virus infection and the acquired immunodeficiency syndrome.* Clinical Infectious Diseases, 1988. **10**(6): p. 1142-1154.
10. Selwyn, P.A., et al., *A prospective study of the risk of tuberculosis among intravenous drug users with human immunodeficiency virus infection.* New England journal of medicine, 1989. **320**(9): p. 545-550.
11. Pitchenik, A.E., et al., *Tuberculosis, atypical mycobacteriosis, and the acquired immunodeficiency syndrome among Haitian and non-Haitian patients in South Florida.* Annals of Internal Medicine, 1984. **101**(5): p. 641-645.
12. Sunderam, G., et al., *Tuberculosis as a manifestation of the acquired immunodeficiency syndrome (AIDS).* Jama, 1986. **256**(3): p. 362-366.
13. Pitchenik, A.E., et al., *Human T-cell lymphotropic virus-III (HTLV-III) seropositivity and related disease among 71 consecutive patients in whom tuberculosis was diagnosed: a prospective study.* American Review of Respiratory Disease, 1987. **135**(4): p. 875-879.
14. Sharma, S.K. and A. Mohan, *Extrapulmonary tuberculosis.* Indian J Med Res, 2004. **120**(4): p. 316-53.

15. Kaya, A., et al., *Pulmonary tuberculosis with multifocal skeletal involvement.* Monaldi Arch Chest Dis, 2004. **61**(2): p. 133-5.

16. Turgut, M., *Spinal tuberculosis (Pott's disease): its clinical presentation, surgical management, and outcome. A survey study on 694 patients.* Neurosurgical review, 2001. **24**(1): p. 8-13.

17. Garg, R.K. and D.S. Somvanshi, *Spinal tuberculosis: a review.* J Spinal Cord Med, 2011. **34**(5): p. 440-54.

18. Schirmer, P., C.A. Renault, and M. Holodniy, *Is spinal tuberculosis contagious?* Int J Infect Dis, 2010. **14**(8): p. e659-66.

19. Flamm, E.S., *Percivall Pott: an 18th century neurosurgeon.* J Neurosurg, 1992. **76**(2): p. 319-26.

20. Batirel, A., et al., *The course of spinal tuberculosis (Pott disease): results of the multinational, multicentre Backbone-2 study.* Clin Microbiol Infect, 2015. **21**(11): p. 1008 e9-1008 e18.

21. Dunn, R., I. Zondagh, and S. Candy, *Spinal tuberculosis: magnetic resonance imaging and neurological impairment.* Spine (Phila Pa 1976), 2011. **36**(6): p. 469-73.

22. Jutte, P.C. and J.H. Van Loenhout-Rooyackers, *Routine surgery in addition to chemotherapy for treating spinal tuberculosis.* Cochrane Database Syst Rev, 2006(1): p. Cd004532.

23. Modic, M., et al., *Vertebral osteomyelitis: assessment using MR.* Radiology, 1985. **157**(1): p. 157-166.

24. Moorthy, S. and N.K. Prabhu, *Spectrum of MR imaging findings in spinal tuberculosis.* American Journal of Roentgenology, 2002. **179**(4): p. 979-983.

25. Shetty, A., R.M. Kanna, and S. Rajasekaran. *TB spine—Current aspects on clinical presentation, diagnosis, and management options*. in *Seminars in Spine Surgery*. 2016. Elsevier.

26. Altini, C., et al., *Comparison of the Diagnostic Value of MRI and Whole Body (18)F-FDG PET/CT in Diagnosis of Spondylodiscitis.* J Clin Med, 2020. **9**(5).

27. Burel, J.G., et al., *Host Transcriptomics as a Tool to Identify Diagnostic and Mechanistic Immune Signatures of Tuberculosis.* Frontiers in Immunology, 2019. **10**(221).

28. Penn-Nicholson, A., et al., *RISK6, a 6-gene transcriptomic signature of TB disease risk, diagnosis and treatment response.* Scientific Reports, 2020. **10**(1): p. 8629.

29. Zak, D.E., et al., *A blood RNA signature for tuberculosis disease risk: a prospective cohort study.* Lancet, 2016. **387**(10035): p. 2312-2322.

30. Suliman, S., et al., *Four-Gene Pan-African Blood Signature Predicts Progression to Tuberculosis.* Am J Respir Crit Care Med, 2018. **197**(9): p. 1198-1208.

31. Thompson, E.G., et al., *Host blood RNA signatures predict the outcome of tuberculosis treatment.* Tuberculosis, 2017. **107**: p. 48-58.

32. Ault, R.C., et al., *Blood RNA signatures predict recent tuberculosis exposure in mice, macaques and humans.* Scientific Reports, 2020. **10**(1): p. 16873.

33. Schlesinger, N., et al., *Tuberculosis of the spine: experience in an inner city hospital.* J Clin Rheumatol, 2005. **11**(1): p. 17-20.

34. Ozol, D., A. Koktener, and M.E. Uyar, *Active pulmonary tuberculosis with vertebra and rib involvement: case report.* South Med J, 2006. **99**(2): p. 171-3.

35. Sankaran, B., *Tuberculosis of bones and joints.* Ind J Tub, 1993. **40**: p. 109-18.

36. Pertuiset, E., et al., *Spinal tuberculosis in adults. A study of 103 cases in a developed country, 1980-1994.* Medicine (Baltimore), 1999. **78**(5): p. 309-20.

37. Ansari, S., et al., *Pott's Spine: Diagnostic Imaging Modalities and Technology Advancements.* N Am J Med Sci, 2013. **5**(7): p. 404-11.

38. Agrawal, V., P.R. Patgaonkar, and S.P. Nagariya, *Tuberculosis of spine.* J Craniovertebr Junction Spine, 2010. **1**(2): p. 74-85.

39. Modi, G., et al., *Non-traumatic myelopathy at the Chris Hani Baragwanath Hospital, South Africa--the influence of HIV.* Qjm, 2011. **104**(8): p. 697-703.

40. Candy, S., G. Chang, and S. Andronikou, *Acute myelopathy or cauda equina syndrome in HIV-positive adults in a tuberculosis endemic setting: MRI, clinical, and pathologic findings.* AJNR Am J Neuroradiol, 2014. **35**(8): p. 1634-41.

41. Zinn, C., M. Vorster, and M.M. Sathekge, *Spinal tuberculosis evaluated by means of 18F-FDG PET/CT: Pilot study.* The Open Nuclear Medicine Journal, 2014. **6**(1).

42. Fuster, D., et al., *Prospective comparison of whole-body (18)F-FDG PET/CT and MRI of the spine in the diagnosis of haematogenous spondylodiscitis.* Eur J Nucl Med Mol Imaging, 2015. **42**(2): p. 264-71.

43. Bassetti, M., et al., *Higher fluorine-18 fluorodeoxyglucose positron emission tomography (FDG-PET) uptake in tuberculous compared to bacterial spondylodiscitis.* Skeletal Radiol, 2017. **46**(6): p. 777-783.

44. Advani, J., et al., *Whole Genome Sequencing of Mycobacterium tuberculosis Clinical Isolates From India Reveals Genetic Heterogeneity and Region-Specific Variations That Might Affect Drug Susceptibility.* Frontiers in Microbiology, 2019. **10**(309).

45. Faksri, K., et al., *Comparative whole-genome sequence analysis of Mycobacterium tuberculosis isolated from tuberculous meningitis and pulmonary tuberculosis patients.* Scientific Reports, 2018. **8**(1): p. 4910.
